# Tri–Band and Dual–Response Smart Windows With Asymmetric Mid–Infrared Emissivity for Year–Round Energy Conservation

**DOI:** 10.1002/advs.202511076

**Published:** 2026-02-24

**Authors:** Cheng Wang, Junwei Liu, Zhihua Zhou, Yuechao Chao, Xueqing Yang, Yan Liang, Yifan Zhou, Weiyi Zhang, Yahui Du, Wufan Wang, Shuqi Zhang, Jinyue Yan

**Affiliations:** ^1^ School of Environmental Science and Engineering Tianjin University Tianjin China; ^2^ Department of Building Environment and Energy Engineering The Hong Kong Polytechnic University Kowloon Hong Kong China; ^3^ International Centre of Urban Energy Nexus The Hong Kong Polytechnic University Kowloon Hong Kong China

**Keywords:** asymmetric emissivity, photochromic, smart window, solar regulation, thermochromic

## Abstract

Building energy consumption accounts for 20%–40% of global energy usage, with windows among the least energy–efficient components. For this issue, thermochromic smart windows have emerged as a cost–effective, stimuli–responsive approach to enhance building energy performance. However, achieving long–term improvements in the indoor lighting environment remains a significant challenge for this technology. In this work, we developed a dual–responsive hydrogel that combines photochromic and thermochromic functionalities, exhibiting excellent shrinkage resistance, tunable spectral characteristics, and robust color–changing behavior. By integrating these hydrogels with solar selective and indium tin oxide films, we designed a tri–band regulation smart window featuring asymmetric mid–infrared emissivity, capable of simultaneously managing visible and near–infrared light to effectively reduce indoor heat gain and loss. The resulting smart window demonstrated superior spectral performance, with a solar modulation rate (*ΔT_sol_*) of 40.8%, visible light modulation (*ΔT_lum_*) of 61.4%, near–infrared blocking of 71.7%, and an emissivity contrast between the inner and outer surfaces (*Δε*) of 66%. These outstanding properties translate into annual energy savings of 14.2%–24.4% for curtain wall buildings globally, excluding polar regions. The concept of tri–band dual–response regulation significantly enhances the energy saving potential of smart windows and broadens their prospects for practical application.

## Graphical abstract

Our promising smart window exhibits exceptional optical properties, enabling the indoor temperature reduction of 12°C during summer and delivering the annual energy savings of 14.2%–24.4% across mid–to–low latitudes.

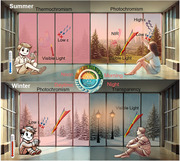

## 1 Introduction

Climate change has emerged as a pressing global challenge. Under the Paris Agreement, the Intergovernmental Panel on Climate Change (IPCC) established a 1.5°C global warming target, highlighting the urgent need to reduce energy consumption and carbon emissions [1, 2, 3, 4]. In this context, the building sector has been widely recognized as a critical area for mitigation efforts, accounting for 20%–40% of total energy use [5, 6, 7]. Within buildings, heating, ventilation, and air conditioning (HVAC) systems contribute to 30%–50% of energy consumption [8, 9, 10]. Reducing HVAC–related energy use thus requires lowering the building's heating and cooling loads, which are strongly influenced by building envelopes. Among building components, windows are widely recognized as the least energy–efficient elements of the envelope, potentially responsible for more than 30% of heat gain or loss [11, 12, 13]. Consequently, the development and deployment of energy–efficient windows are essential for mitigating global warming and addressing energy shortages.

Thermochromic smart windows, which modulate transparency in response to ambient temperature, are widely regarded as a cost–effective and environmentally friendly approach to improving building energy efficiency [14, 15, 16, 17, 18]. A variety of thermochromic materials, including VO_2_ [19, 20], perovskites [21, 22], hydrogels [23, 24, 25, 26], organic liquid crystals [27, 28], and ionic liquids [29, 30, 31], have garnered significant research attention worldwide. In particular, thermochromic hydrogels have been extensively developed and optimized due to simple fabrication process, suitable phase transition temperatures, and excellent solar modulation capabilities. The incorporation of 4% ethanol into poly(N–isopropylacrylamide) (PNIPAM) hydrogels has been demonstrated to elevate the solar modulation rate to 71.8%, accompanied by an indoor temperature reduction of 14.5°C [32]. Meanwhile, the integration of inorganic salts into PNIPAM hydrogels enables a downward adjustment of phase transition temperature to below 30°C, achieving a glass surface temperature reduction of more than 3.5°C [33, 34]. Despite these advances, most thermochromic hydrogels suffer from poor mechanical stability and shrinkage at high temperatures, limiting their long–term performance in building windows. Moreover, these hydrogels typically exhibit abrupt switching between colorless–transparent and white–opaque states, which restricts their ability to regulate the indoor light environment and may compromise visual comfort.

Photochromic technology, which enables color change in response to light exposure, offers a promising strategy to overcome the limitations of conventional thermochromic systems in regulating indoor lighting [35, 36, 37]. The doping of Li ions into tungsten oxide (WO_3_) matrices has been shown to yield photochromic smart windows capable of reversible transformation from a colorless state (*T_lum_* = 80%) to a green state (*T_lum_* = 30%) under LED light [38]. Furthermore, the incorporation of W_18_O_49_ nanoparticles into PNIPAM hydrogels has enabled the fabrication of dual–responsive photochromic–thermochromic composites, which exhibit a solar modulation rate of 67.4% and can reduce indoor temperatures by 8.5°C [39]. Despite these achievements, both photochromic and thermochromic materials generally neglect the effects of near–infrared and mid–infrared spectra, thereby limiting their potential for year–round energy savings.

**FIGURE 1:**
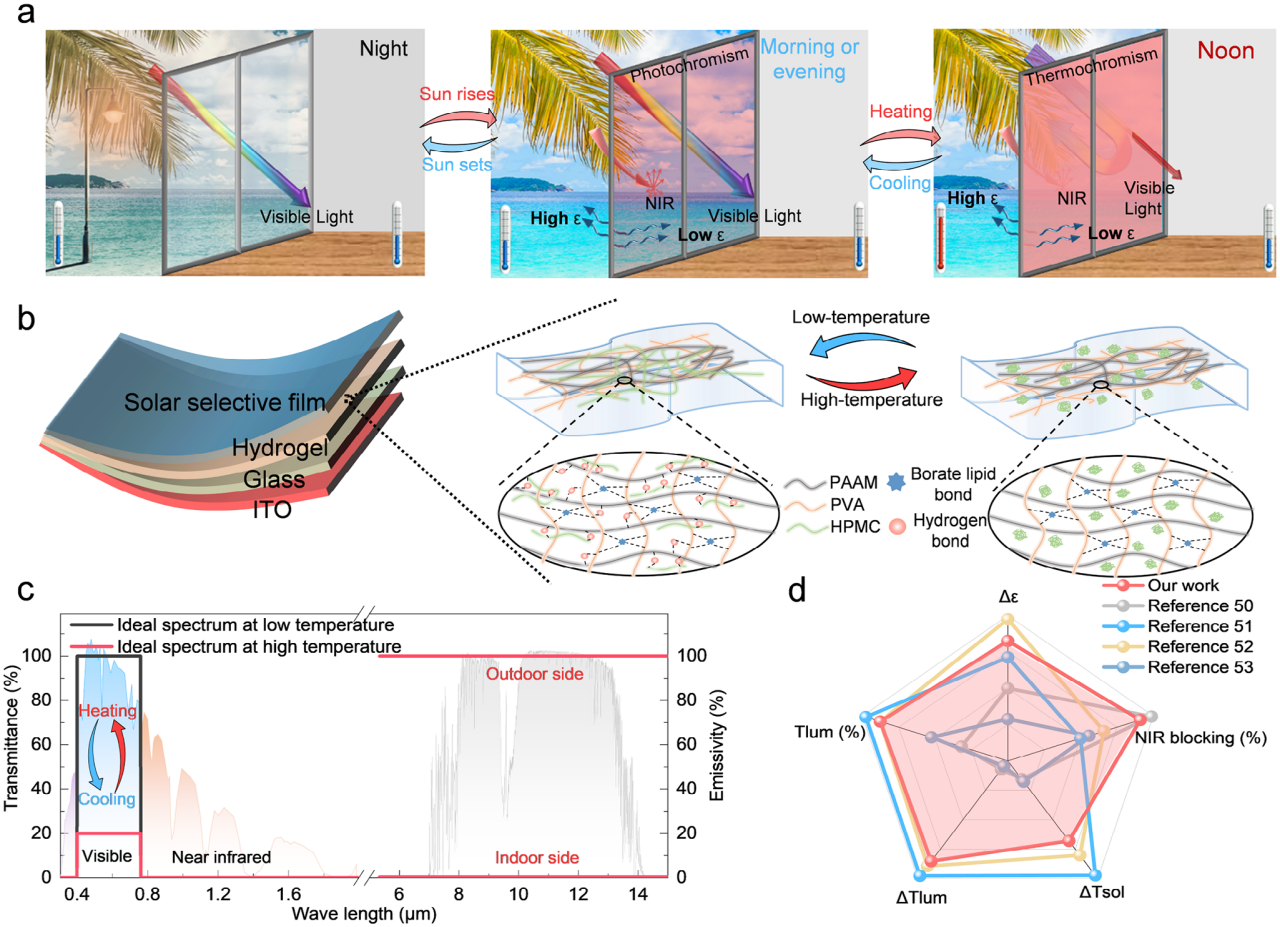
Basic concept and properties of AMDTR smart windows. (a) The working principle of AMDTR smart windows. (b) Structure design of AMDTR smart windows. (c) Ideal spectral curve of AMDTR smart windows. (d) The advantages in spectral properties of AMDTR smart windows prepared in this work [50, 51, 52, 53].

The integration of solar selective and radiative cooling technology offers a promising strategy to overcome the limitations of conventional thermochromic smart windows and enhance their energy saving potential [40, 41]. Solar selective materials reduce summer heat gain by blocking near–infrared radiation, whereas radiative cooling dissipates heat through the atmospheric window (8–13 µm) to outer space [42, 43, 44, 45, 46, 47, 48, 49 ]. Nevertheless, solar selective glazing may absorb near–infrared light and re–transfer heat indoors, while radiative cooling will exacerbate winter heat loss by enhancing indoor–outdoor heat exchange. Introducing asymmetric mid–infrared emissivity between the inner and outer window surfaces (i.e., asymmetric mid–infrared emissivity) can mitigate summer heat gain and suppress winter heat loss [49]. Therefore, developing novel materials and structures for smart windows to optimize their spectral properties is highly desirable for further improving their annual energy saving performance (Figure 1c).

In this work, we proposed a novel approach to enhance the long–term stability, spectral properties, and coloration ability of smart windows, providing new insights for improving their energy saving performance and practicality. Specifically, we first developed a dual–responsive hydrogel that integrates photochromic and thermochromic functionalities, yielding excellent mechanical robustness, tunable spectral properties, and controllable coloration to address challenges related to durability and indoor visual comfort. We then developed a smart window with asymmetric emissivity (AMDTR smart window) by integrating the hydrogels, solar selective films and ITO films (Figure 1a,b), achieving top–notch spectral characteristics (Figure 1d). Furthermore, we examined the color changes of AMDTR smart window to assess its practical application potential in buildings. Outdoor experiments were conducted to examine its summer cooling performance, demonstrating the combined benefits of tri–band spectral management and asymmetric mid–infrared emissivity. Finally, we performed the simulations across different climate zones, emphasizing the energy saving advantages of AMDTR smart window over other energy–efficient window technologies. Overall, the design of tri–band, dual–responsive smart windows with asymmetric mid–infrared emissivity provides a significant advance toward high–performance, energy saving smart window systems.

## 2 Results and Discussion

### 2.1 Design and Basic Properties of Dual–Responsive Hydrogels

**FIGURE 2:**
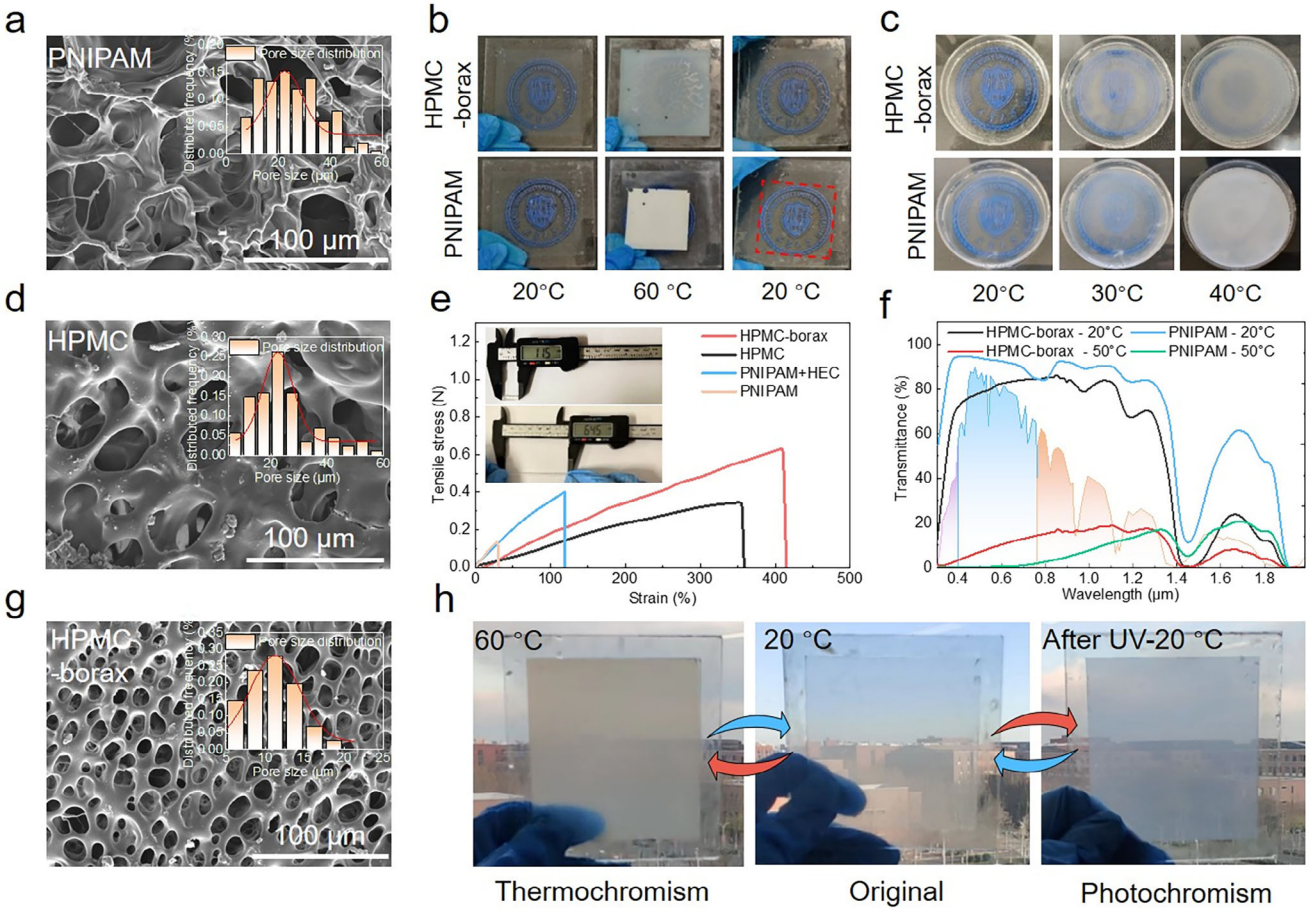
Design and basic properties of dual–responsive hydrogels. Pore size distribution of PNIPAM (a), HPMC (d) and HPMC–borax hydrogels (g). (b) Anti–shrinkage of HPMC–borax hydrogels. (c) Thermochromic effect of HPMC–borax hydrogels. (e) Mechanical properties of HPMC–borax hydrogels. (f) Spectral properties of HPMC–borax hydrogels. (h) Photochromic and thermochromic images of the hydrogels.

To obtain thermochromic hydrogels with superior mechanical strength and shrinkage resistance, borax was incorporated into hydroxypropyl methylcellulose (HPMC) hydrogels using a simple solution–based preparation method (Notes S1 and S2). To confirm successful polymerization, Fourier transform infrared (FTIR) spectra was employed to detect characteristic bands for three hydrogels (Figure S1). After the addition of borax, the hydrogel exhibited stretching vibration peak at 1261 cm^−^^1^, indicating the formation of borate ester bonds and resulting in a more compact network structure. Scanning electron microscopy (SEM) analysis revealed a significant reduction in the pore size of the HPMC–borax hydrogels (Figure 2a,d,g). The PNIPAM hydrogel exhibited an average pore size of 28.54 µm, and the HPMC hydrogel showed 24.5 µm; in contrast, the pore size of the HPMC–borax hydrogel decreased substantially to 11.6 µm. This structural advantage allowed the HPMC–borax hydrogels to achieve a tensile strength of 0.615 N and endure over 400% deformation, significantly surpassing the performance of HPMC hydrogels and the widely used PNIPAM hydrogels (Figure 2e).

Notably, unlike PNIPAM hydrogels which undergo pronounced shrinkage under elevated temperatures, the HPMC–borax hydrogels exhibited negligible volume change after repeated thermal cycling between 20°C and 60°C (Figure 2b). This stability is primarily attributed to the temperature–independent hydrophilicity of the structural support network and the presence of borate ester bonds, which effectively suppress thermally induced contraction. The HPMC–borax hydrogel exhibited remarkable anti–shrinkage even under environmental exposure (Figure S2), demonstrating its potential for shading applications in architectural windows. Encouragingly, after 100 heating–cooling cycles, the hydrogel retained outstanding light modulation performance and anti–shrinkage properties without significant degradation, demonstrating its long–term stability for window applications (Figure S3).

To further evaluate the solar modulation capability of the HPMC–borax hydrogels, we examined their temperature–dependent spectral regulation. The results indicated that HPMC–borax hydrogel exhibited a visible light transmittance of 80.8% at 20°C, with a solar modulation rate (*ΔT_sol_*) of 63.1% and a visible light modulation rate (*ΔT_lum_*) of 71.4%, demonstrating the exceptional solar regulation and cooling potential (Figure 2f). Notably, the HPMC–borax hydrogel exhibited a transition temperature similar to that of PNIPAM, supporting its application in tropical regions such as Hong Kong (Figure S4). In contrast to PNIPAM, it displayed a gradual color transition while retaining partial transparency even at high temperatures (Figure 2c). This property not only satisfies the indoor natural lighting requirements but also helps reduce the building's lighting energy consumption. Collectively, these attributes highlight the exceptional potential of the HPMC–borax hydrogels for advanced solar control applications.

Given that thermochromic hydrogels have limitations in meeting visual comfort for indoor occupants, we incorporated ultraviolet−responsive photochromic materials into the HPMC–borax hydrogel. Since excessive photochromic powder can aggregate and reduce transparency, we first optimized its concentration for desirable optical performance. The hydrogels with 0.1% concentrations of photochromic powders achieved satisfactory transparency and distinct color change (Figures S5–S7). Promisingly, the photochromic powders did not substantially interfere with the thermochromic properties of the hydrogel matrix, demonstrating the successful integration of photochromic technology in regulating indoor lighting environments (Figure 2h).

### 2.2 Spectral Characteristics of AMDTR Smart Windows

**FIGURE 3:**
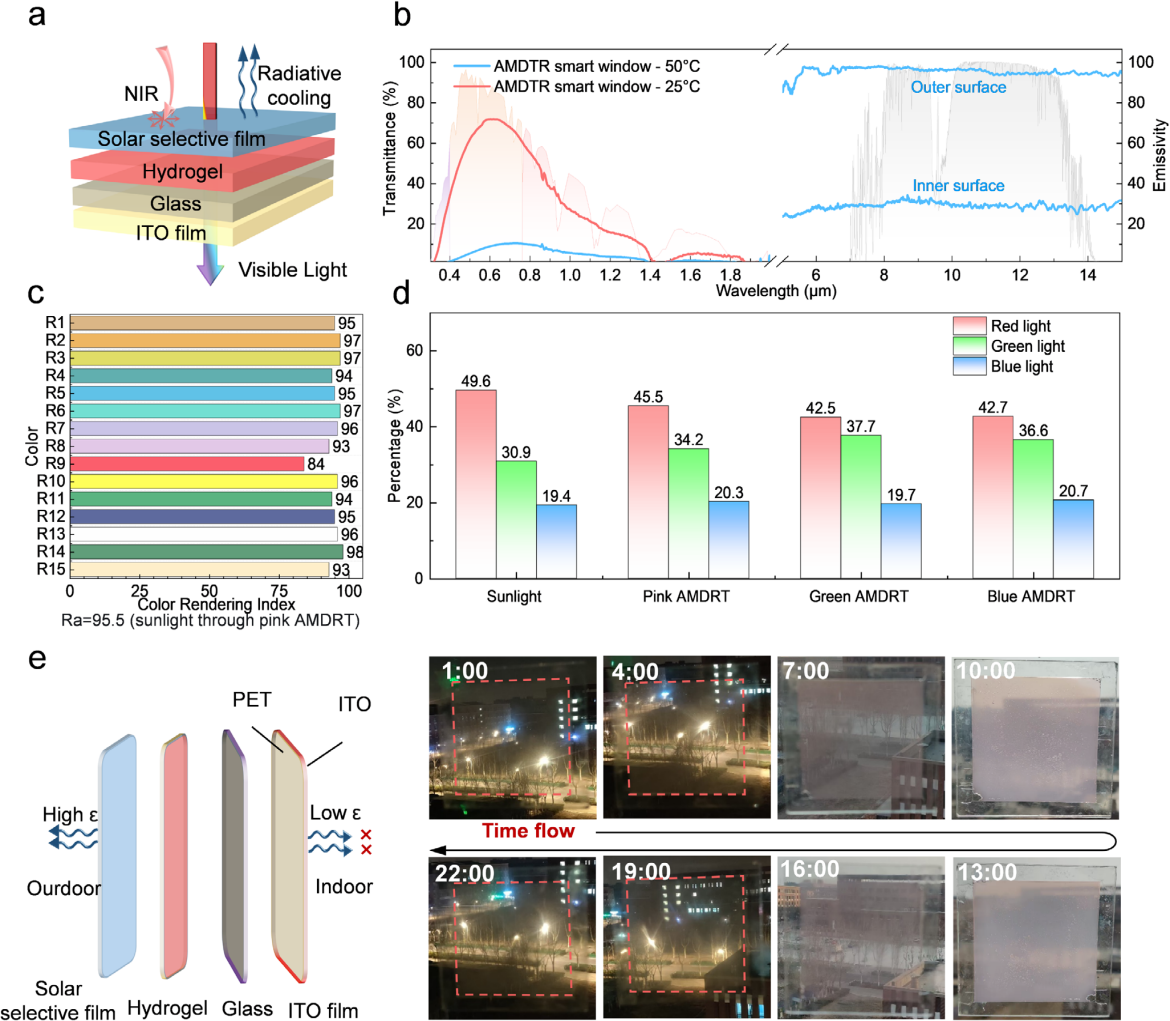
Spectral characteristics and practicality of AMDTR smart windows. (a) The structure diagram of AMDTR smart windows. (b) The spectral characteristics of AMDTR smart windows. (c) Color rendering index of sunlight transmitted through the pink AMDRT smart window. (d) RGB color ratios of sunlight through different AMDRT smart windows (e) The color change process of AMDTR smart windows throughout an entire day in summer.

Given that photochromic and thermochromic hydrogels alone have minimal influence on mid–infrared emissivity and offer limited regulation in the near–infrared range, additional design strategies are required to further enhance the energy saving performance of dual–responsive hydrogels. Integrating this dual–responsive hydrogel with a solar selective film [54] and an ITO film constitutes a promising design strategy. Crucially, the functional layer sequence of the AMDTR smart window is critical to its radiative cooling performance. We investigated the impact of various configurations on mid–infrared emissivity (Note S2, Figures S8 and S9) and found that placing the solar selective film on the outer surface and ITO on the inner surface yielded the maximum emissivity difference (Figure S10, Tables S1 and S2). This performance is attributed to the high mid–infrared reflectivity of the ITO film (up to 68.8%) and the superior emissivity of the solar selective film (up to 94.8%). Analysis confirms that the device's surface emissivity is dominated by the outermost layer (∼99%) rather than the sub–outer layer (∼1%) (Figures S11 and S12). Consequently, balancing mid–infrared emissivity with encapsulation feasibility, we identified the optimal structure as solar selective film/hydrogel/glass/ITO (Figure 3a).

Building on the optimization of the functional layer sequence, we integrated the developed dual–responsive hydrogels with a solar selective film, an ITO film, and glass to construct the AMDTR smart window (Note S3). Spectral analysis results indicated that AMDTR smart window exhibited exceptionally high transparency (71.9%) and near–infrared blocking rate (71.7%), with satisfactory visible light modulation (*ΔT_lum_* = 61.4%) and solar modulation rate (*ΔT_sol_* = 40.8%) (Figure 3a,b). Moreover, the mid–infrared emissivity of the inner and outer surfaces of the AMDTR smart window reached 30.1% and 96.1%, respectively, demonstrating the strong potential to block indoor heat gain in summer and heat loss in winter. Notably, compared to previous reports incorporating Cs_0.33_WO_3_ nanoparticles directly into the hydrogels [41], AMDTR smart window can improve the average visible light transmittance by 12.4%, while maintaining similar near–infrared blocking (Figure S13). This improvement arises from the large refractive index mismatch between Cs_0.33_WO_3_ nanoparticles and the aqueous hydrogel matrix, which enhances light scattering and reduces transparency. In contrast, the AMDTR configuration effectively minimizes scattering, thereby enabling a substantial increase in visible transmittance.

Color fidelity of the AMDRT smart window is critical to indoor visual comfort. Therefore, we tested the Color Rendering Index (CRI) of AMDRT smart windows integrated with different photochromic powders (Note S4). Satisfactorily, the CRIs of windows with pink, green, and blue photochromic powders reached 95.5, 91.5, and 92.3, respectively (Figure 3c; Figures S14–S17), confirming that all variants maintain excellent daylighting performance and color fidelity. The pink AMDTR smart window exhibited the highest fidelity, which is likely attributable to the optical properties of commercial glass; standard glass typically transmits more green light while absorbing a significant portion of red light (Figure S18). Consequently, hydrogels that further filter red light compromise fidelity. This is supported by the red, green, and blue (RGB) analysis, where the primary color ratio of the pink AMDTR window most closely resembled that of natural sunlight (Figure 3d). Therefore, the pink AMDTR smart window was selected for subsequent experimental studies.

To further evaluate the practicality of AMDTR smart window, we investigated the actual changes in transparency and color under summer conditions. Obviously, AMDTR smart window demonstrated outstanding transparency at night. By 7 a.m., the UV light in sunlight induced the photochromic effect of hydrogels, while the intensity of sunlight is insufficient to trigger the thermochromic effect. As a result, AMDTR smart window presented the distinct pink transparent state, meeting the color comfort needs of indoor occupants (Figure 3e). By noon, high–intensity sunlight triggered both thermochromic and photochromic effects, turning the AMDTR smart window pink and opaque at noon, thereby achieving excellent cooling effect in summer. Notably, AMDTR smart window switched to a colorless and transparent state after sunset, demonstrating the exceptional cyclic stability in terms of transparency and coloration. In addition, winter ambient temperatures were insufficient to trigger the thermochromic response of the AMDTR smart window, enabling sunlight to penetrate indoors for illumination and passive heating (Figure S19). The window showed slight color change under UV light in midday winter, enhancing indoor visual comfort. Overall, our developed AMDTR smart window demonstrated the environment–adaptive transparency and color tuning to summer and winter conditions.

Given the requirement of long–term service in complex outdoor environments, we further evaluated the wear resistance of AMDTR smart windows (Note S5). After 7500 friction cycles, the outer surface of AMDTR smart windows remained smooth without visible scratches, demonstrating excellent wear resistance (Figure S20). This advantage ensured that the average visible light transmittance of AMDTR smart window only decreased by 1.6% (from 71.9% to 70.3%) post–friction (Figure S21). Beyond its validated anti–shrinkage performance and thermal cycling stability, resistance to photo and thermal fatigue is also critical for practical applications. After one month of actual outdoor cycling, the AMDTR smart window retained excellent thermochromic and photochromic functionalities (Figure S22). Collectively, our AMDTR smart windows exhibited outstanding wear resistance and photo/thermal fatigue resistance, underscoring their broad potential for long–term architectural window applications.

### 2.3 Performance of AMDTR Smart Windows

**FIGURE 4:**
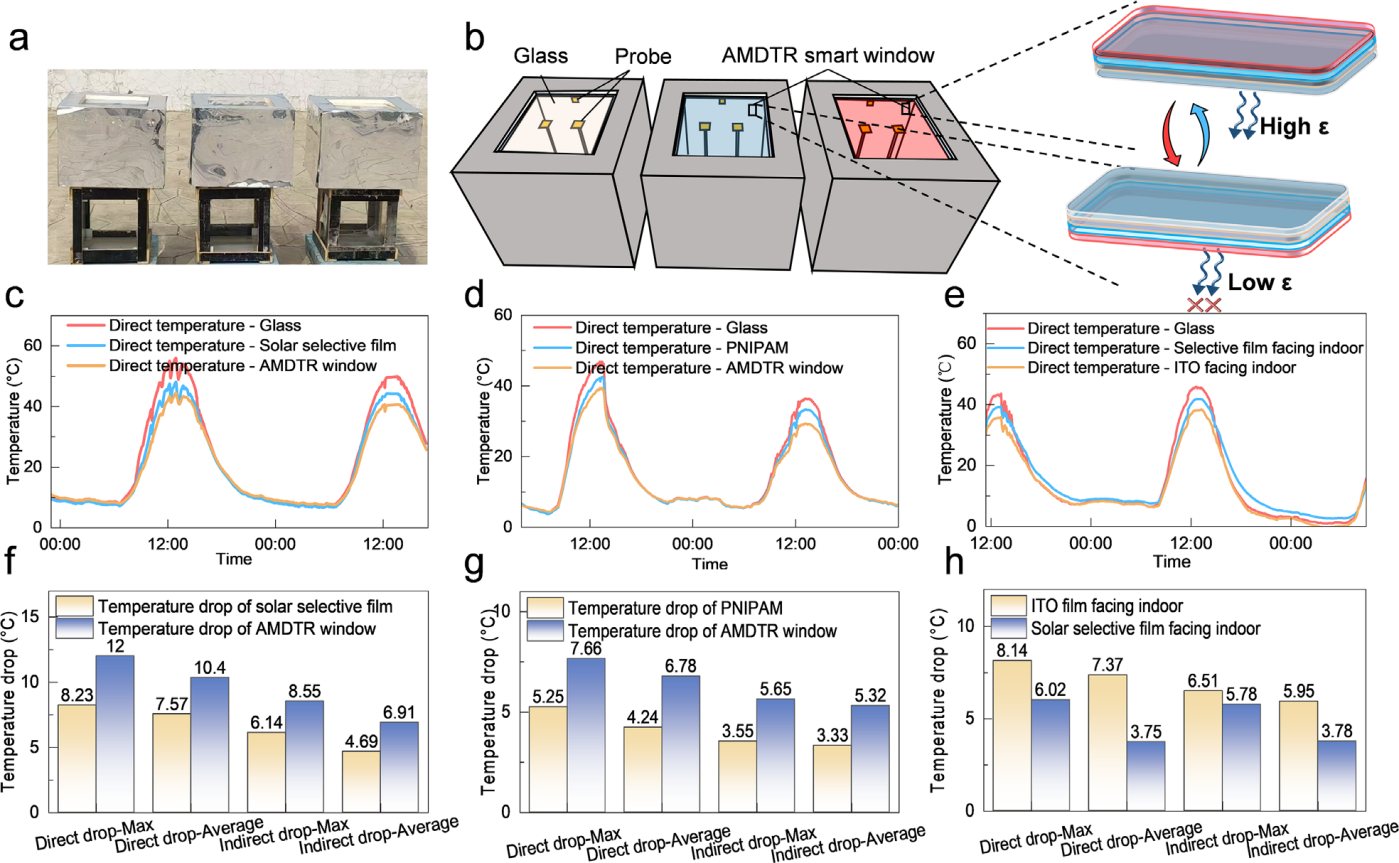
Cooling performance of AMDTR smart windows. The actual image (a) and schematic diagram (b) of cooling experimental devices. Indoor temperature of AMDTR smart windows compared with solar selective films (c) and PNIPAM hydrogels (d). (e) Indoor temperature of AMDTR smart windows with different surfaces facing indoor. Direct and indirect temperature drop of AMDTR smart windows compared with solar selective films (f) and PNIPAM hydrogels (g). (h) Direct and indirect temperature drop of AMDTR smart windows with different surfaces facing indoor.

To further assess the actual cooling performance of AMDTR smart windows, we set up three experimental devices (Figure 4a,b), and measured the indoor temperatures in both sun–exposed areas (direct temperature) and shaded areas (indirect temperature) (Note S7). The results showed our AMDTR smart window reduced direct and indirect temperatures by up to 12°C and 8.55°C, respectively—markedly outperforming conventional solar selective films—validating the energy saving contribution of its thermochromic regulation (Figure S25; Figure 4c,f). Under separate sunny–day conditions, the AMDTR smart window can lower indoor direct temperature by up to 7.66°C, exceeding the 5.25°C reduction from PNIPAM hydrogels (Figure 4d,g). This superior performance stems from the AMDTR smart window's long–term structural stability, whereas PNIPAM hydrogels tend to shrink over time, impairing their ability to effectively block sunlight.

To investigate the effectiveness of asymmetric mid–infrared emissivity in preventing heat gain, we further examined the temperature drop when the high–emissivity side (solar selective film) and low–emissivity side (ITO film) faced indoor (Figure 4b). As anticipated, when the high–emissivity side faces indoor, the corresponding direct and indirect temperature drops reached approximately 3.75°C and 3.78°C (11:30–14:00), respectively. In contrast, when the low–emissivity side faces indoor, AMDTR smart windows can reduce the indoor direct and indirect temperatures by about 7.37°C and 5.95°C (Figure 4e,h), highlighting the potential of low–emissivity surface in reducing indoor heat gain and the corresponding cooling energy consumption. Overall, compared to other energy–efficient window technologies, our AMDTR smart windows can provide superior cooling performance in summer, showcasing their superiority in reducing cooling energy consumption.

### 2.4 Building Energy Saving With AMDTR Smart Windows

**FIGURE 5:**
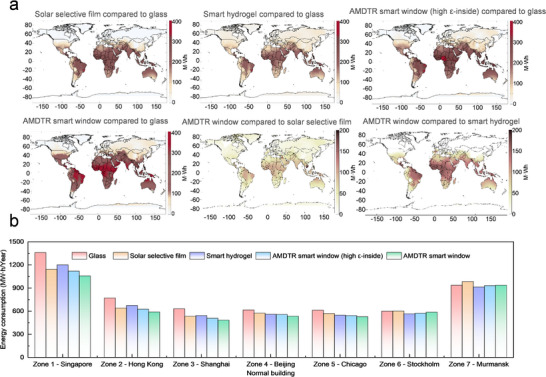
The energy saving potential of different energy saving window technologies in different regions of the world. (a) The energy saving advantages of AMDTR smart windows compared to other energy saving technologies in the world. (b) Annual energy savings of different energy saving window technologies for normal buildings.

To further reveal the superior energy saving performance of AMDTR smart windows across various regions, we compared the energy saving effects of various window technologies for a typical office building (Note S7). The results indicate that most energy–efficient window technologies offer the substantial savings in many regions worldwide (Figure 5a). Solar selective films can provide over 200 MWh of annual energy savings in low–latitude regions; however, in high–latitude regions, they may increase building energy consumption. Conversely, thermochromism hydrogels showed outstanding efficacy in high–latitude regions (e.g., Russia) with annual savings ranging from 11.1 to 60.8 MWh. However, these locations showcased suboptimal performance relative to solar selective films in tropical zones with the annual energy savings below 200 MWh (Figure 5a). By combining the advantages of solar selective and thermochromic technologies, AMDTR smart windows deliver enhanced energy savings and broader applicability across global regions. Simulations indicate that AMDTR smart windows achieved the annual energy savings of over 300 MWh in low–latitude regions, surpassing both standalone solar selective films and thermochromic hydrogels. Additionally, in high–latitude regions, AMDTR smart windows can maintain the markedly improved energy efficiency with the annual energy savings of up to 26.4 MWh. These results are significantly higher than that achieved by solar selective films alone, demonstrating the effectiveness of the low–emissivity on the indoor side in mitigating winter heat loss.

To quantify the global energy saving potential of AMDTR smart windows, we assessed the annual energy consumption of both conventional and glass curtain wall buildings across seven representative cities using different energy–efficient window technologies (Note S8). Compared to conventional buildings in mid–to low–latitude regions, including Singapore, Hong Kong, Shanghai, Beijing, and Chicago, AMDTR smart windows can reduce annual energy consumption by 10.6%–23.5%, outperforming solar selective films (6.6%–17.1%) and thermochromic hydrogels (9.2%–14.0%) (Figure 5b). Compared to glass curtain buildings in the mid–to low–latitude regions, AMDTR smart windows can achieve the impressive annual energy savings of 14.2%–24.4%, significantly exceeding solar selective films (9.8%–21.1%) and thermochromic hydrogels (12.1%–16.7%). On the other hand, in high–latitude regions (such as Stockholm and Murmansk), AMDTR smart windows can reduce the annual building energy consumption by 0.6%–2.1% for normal buildings and by 1.5%–9.9% for glass curtain wall buildings (Figure 5b; Figure S29). This is primarily attributed to the heat retention effect of low–emissivity on the indoor side in cold environments. These results demonstrate that AMDTR smart windows can effectively lower cooling energy demand in summer while mitigating winter heat loss, yielding the exceptional annual energy savings across a wide range of climates and building types.

## 3 Conclusions

In summary, we developed a dual–responsive hydrogel combining photochromic and thermochromic functionalities, achieving excellent spectral performance, mechanical robustness, and reversible color–changing behavior. This innovation overcomes the limitations of conventional hydrogels in regulating indoor lighting. Furthermore, we fabricated AMDTR smart windows via the integration of hydrogels, solar selective films, ITO films, and glass, enhancing year–round energy saving performance. The hydrogels demonstrated negligible shrinkage under the prolonged high temperatures and withstood deformations exceeding 400%. AMDTR smart windows also demonstrated exceptional optical properties, including a visible light transmittance of 72%, a solar modulation rate of 40.8%, a near–infrared blocking rate of 71.7%, and an emissivity difference between the inner and outer surfaces (*Δε*) of 66%. Moreover, our designed smart windows can undergo the periodic color change under sunlight, revealing its significant potential in enhancing occupant color comfort. These features enabled the AMDTR smart windows to reduce indoor temperatures by up to 12°C during summer. In mid–to low–latitude regions, they achieved annual energy savings of 14.2%–24.4% in glass curtain–wall buildings, demonstrating both high energy efficiency and broad applicability. Moving forward, the design of AMDTR smart windows holds great promise for diverse applications, including building facades, vehicles, and greenhouse cultivation.

## Author Contributions

C.W., J.L., and J.Y. conceived the idea and designed the experimental protocols. C.W. performed the fabrication of hydrogels with the assistance of Y.C., X.Y., S.Z., and Y.D. C.W. performed the spectral analysis under the direction of Y.L., Y.Z., W.Z., and W.W. C.W. analyzed the data and wrote the manuscript under the guidance of Z.Z. and J.Y. J.L. and J.Y. directed the research. All authors contributed to the editing of this manuscript.

## Conflicts of Interest

The authors declare no conflicts of interest.

## Supporting information




**Supporting File**: advs74471‐sup‐0001‐SuppMat.docx

## Data Availability

The data that support the findings of this study are available from the corresponding author upon reasonable request.
