# Supplementary material for: Tri–Band and Dual–Response Smart Windows With Asymmetric Mid–Infrared Emissivity for Year–Round Energy Conservation
Source: Adv Sci (Weinh). 2026 Feb 24;13(24):e11076. doi: 10.1002/advs.202511076 (PMC13115933; doi:10.1002/advs.202511076)
Supplement: Supplementary file 1 — Supporting File: advs74471‐sup‐0001‐SuppMat.docx [file ADVS-13-e11076-s001.docx]

*Supporting Information*

**Tri-band and** **Dual-response Smart Windows** **with Asymmetric Mid-infrared Emissivity for Year-Round Energy Conservation**

*Cheng Wang, Junwei Liu*, Zhihua Zhou, Yuechao Chao, Xueqing Yang, Yan Liang, Yifan Zhou,* *Weiyi Zhang, Yahui Du, Wufan Wang, Shuqi Zhang, and Jinyue Yan**

C. Wang, Prof. Z. Zhou, Y. Chao, X. Yang, Y. Du, W. Wang, and S. Zhang

School of Environmental Science and Engineering, Tianjin University, Tianjin, 300350, China.

C. Wang, Dr. J. Liu, Y. Chao, X. Yang, Y. Liang, Y. Zhou, W. Zhang, Y. Du and Prof. J. Yan

Department of Building Environment and Energy Engineering, The Hong Kong Polytechnic University, Kowloon, Hong Kong, China.

Email: junweei.liu@polyu.edu.hk; j-jerry.yan@polyu.edu.hk

Dr. J. Liu, Y. Liang, Y. Zhou, W. Zhang, and Prof. J. Yan

International Centre of Urban Energy Nexus, The Hong Kong Polytechnic University, Kowloon, Hong Kong, China.

# Note S1: Material

Fluorolefin copolymers with alkyl vinyl ethers (FEVE fluorocarbon resin) (model: HK-2X) and curing agent (Isocyanate) were purchased from Jining Huakai Resin Co., Ltd. Cesium tungstate nanoparticles (Cs0.33WO3 nanoparticles) were provided by Shanghai Yingcheng New Materials Co., Ltd. Ethyl alcohol was purchased from Tianjin Yuanli Chemical Co., Ltd. Xylene was purchased from Shanghai Titan Technology Co., Ltd. Polyvinylpyrrolidone dispersant (PVP) was provided by Tianjin Huasheng Chemical Reagent Co., Ltd. Thickening agent (JS) was purchased from Guangdong Nanhui New Materials Co., Ltd. Hydroxypropyl methylcellulose (HPMC) was supplied by Shanghai Macklin Biochemical Co., Ltd. Sodium hydroxide (NaOH) was obtained from Yantai Shuangshuang Chemical Co., Ltd., and polyvinyl alcohol (PVA) was provided by Wuxi Yatai United Chemical Co., Ltd. Acrylic acid was supplied by Tianjin Yuanli Chemical Co., Ltd. Sodium tetraborate (borax) was obtained from Tianjin Chemical Reagent Co., Ltd. ITO films were provided by Shenzhen Siyi Industrial Products Co., Ltd. Photochromic powder was sourced from Shenzhen Magic Color Technology Co., Ltd.

# Note S2: The emissivity of AMDTR smart Windows with different structures

To determine the final AMDTR smart window structure, we further simulated the emissivity of configurations 1–4 (**Figure S8**). Notably, long-term exposure of the hydrogel to air may cause dehydration. Thus, the hydrogel needs to be encapsulated inside the AMDTR smart window. Since the solar selective film, ITO film, and glass effectively block air and water, no additional auxiliary layers are required. The mid-infrared emissivity and reflectance of each functional layer are presented in **Figure S9**. Using these data, we first simulated the inner and outer surface emissivity of the AMDTR smart window under different configurations. We then calculated the cumulative absorption of 8–13 μm mid-infrared radiation by each functional layer, followed by data normalization to investigate the contribution ratio of each layer to emissivity.

# Note S3: Preparation of the AMDTR smart window

**(1) Preparation of solar selective films**

The preparation method of the solar selective film was similar to that described in our previous work.^[1]^ Specifically, 25 g of ethanol was mixed with 20 g of xylene, followed by the addition of 0.5 g of PVP dispersant to the ethanol/xylene mixture. The solution was stirred until the PVP was completely dissolved. Subsequently, 5 g of Cs_0.33_WO_3_ nanoparticles were added, and the mixture was stirred for 10 minutes. The mixture was then ultrasonically dispersed for 15 minutes using an ultrasonic processor (Model: XU-JY88-II), resulting in a nanoparticle precursor solution with a mass concentration of 10%. Subsequently, 4 g of the precursor solution was gradually added to 45 g of FEVE fluorocarbon resin and stirred for 15 minutes using a magnetic stirrer. Following this, 5 g of curing agent and 0.25 g of JS were sequentially added and stirred for an additional 5 minutes. The resulting solution was then coated onto a release film using a wire-bar coater and cured at room temperature for 24 hours, yielding a solar selective film with thickness of 100 µm and Cs_0.33_WO_3_ mass concentration of 0.75%.

**(2) Preparation of thermochromic hydrogels**

Initially, 7 g of HPMC was dissolved in 50 g of deionized water to prepare an HPMC solution. Subsequently, 20 g of the prepared HPMC solution was mixed with 0.25 g of borax, 3 g of acrylic acid, and 6 g of 8.2% NaOH solution. The mixture was stirred for 30 minutes to ensure the complete dissolution of the borax. Following this, 0.06 g of crosslinker, 0.1 g of photoinitiator and 5 g of polyvinyl alcohol solution (mass concentration is 10%) were sequentially added to the mixture, with stirring continued for an additional 30 minutes to ensure full dissolution of all solids and the removal of air bubbles. Lastly, 10 g of additional HPMC solution, 0.075g of photochromic powder, 6 g of acrylic acid and 6 g of the 8.2% NaOH solution were added to the resulting mixture and stirred for 30 minutes, producing the precursor solution for the HPMC-borax hydrogel. Subsequently, the prepared precursor solution was poured into a 1 mm-thick glass interlayer and cooled in 0 ℃ cold water for 10 minutes. Finally, the precursor solution was exposed to UV light for 10 minutes, resulting in the formation of the HPMC-borax hydrogel.

**(3) Preparation of AMDTR smart window**

First, the solar selective film, ITO film, and glass were wetted with deionized water. Subsequently, the solar selective film was adhered to the upper layer of the HPMC-borax hydrogel, while the glass was attached to its bottom layer. Finally, the ITO film was placed on the bottom of the glass, with air bubbles at the interfaces carefully removed to ensure tight interlayer adhesion, thereby forming the target AMDTR smart window. Notably, the ITO film is fabricated by depositing ITO nanoparticles onto a PET substrate via magnetron sputtering; during attachment to the glass, the PET side of the film is bonded to the glass, with the ITO nanoparticle layer facing outward.

# Note S4: Material characterizations

The transmittance spectra of the materials in the 0.3-2 μm range were measured using Ultraviolet-visible-near infrared spectroscopy (UV-VIS-NIR, UV 3600 Plus, Shimadzu, Japan). The emittance in the infrared region (4-25 μm) was determined by Fourier transform infrared spectroscopy (FTIR, Nicolet iS50, Thermo Fisher, USA). The surface morphology was analyzed using field emission scanning electron microscopy (FE-SEM, Quanta 250 FEG, Thermo Fisher, USA). The molecular structure of the film was characterized using FTIR spectroscopy (PerkinElmer).

The Color Rendering Index (CRI) was tested using a wireless spectral illuminometer (Model: BN330). First, the illuminometer was placed under sunlight to measure the CRI of natural light as a control. Subsequently, the surrounding area was sealed with polystyrene foam boards to block ambient scattered light; the pink, green, and blue AMDRT smart windows were then placed above the illuminometer separately to measure the CRI of sunlight transmitted through the windows, thereby evaluating indoor daylighting comfort.

# Note S5 Wear resistance test

In the long-term application of the AMDTR smart window in buildings, the solar selective film on its outer surface inevitably undergoes abrasion by outdoor sand and dust, making wear resistance crucial for its long-term performance. Therefore, a steel ball wrapped in polyester fabric was used as the abrasive counterpart under a 1 N normal force, with reciprocating motion at 1 Hz for 7500 cycles. Abrasion resistance was evaluated by comparing surface morphology and optical transparency before and after testing.

# Note S6: Cooling performance test

To evaluate the practical cooling performance of the developed AMDTR smart window, three test chamber were constructed (**Figure S23**), and cooling experiments were conducted on the rooftop of the School of Environmental Science and Engineering at Tianjin University (39.1257°N, 117.2273°E). The internal dimensions of each test chamber were 30 cm × 30 cm × 25 cm, with walls and bottom made of 8 mm-thick polystyrene foam boards to minimize heat transfer. The exterior surfaces of the test chambers were covered with mirrored aluminum to reduce solar radiation heating. The top of the first test chamber was consistently fitted with plain glass as the control group. The second test chamber was permanently equipped with the AMDTR smart window, with its high-emissivity surface facing outdoors and low-emissivity surface facing indoors. For the third test chamber, a solar selective film, PNIPAM hydrogel, and the AMDTR smart window (with its high-emissivity surface facing indoors) were alternately placed during different experimental phases to demonstrate the superiority of the AMDTR smart window with the low-emissivity surface facing indoors. Temperature measurement points were set in respectively in direct sunlight and shaded areas inside the experimental boxes. Among them, the measurement points exposed in direct sunlight were covered with light-yellow fabric to simulate the surface temperature of human skin. The measuring points in the shade were used to measure the indoor temperature.

# Note S7: Energy consumption simulation

The actual energy-saving performance of the prepared AMDTR smart window is influenced by building type and meteorological parameters. Therefore, in this work, we developed thermal balance models for windows, walls and indoor air to calculate the annual energy consumption of air-conditioning and lighting (**Note S8**). The models were applied to two typical office building types: normal buildings and glass curtain wall buildings **(Figure S24 and Table S3)**. Subsequently, for typical normal office buildings, we investigated the global energy savings of the the AMDTR smart window compared to other types of energy-efficient windows, highlighting the application potential of the prepared AMDTR smart window. Finally, we selected 7 representative cities from seven climate zones, to demonstrate the energy-saving superiority of the AMDTR smart window compared to other energy-efficient windows under varying climatic conditions. The selected cities were Singapore (Zone 1), Hong Kong (Zone 2), Shanghai (Zone 3), Beijing (Zone 4), Chicago (Zone 5), Stockholm (Zone 6), and Murmansk (Zone 7).

# Note S8: Thermal/optical transfer model for buildings

(1) Thermal equilibrium of the film surface:

$\text{Q}_{\text{solar}}\text{+}\text{Q}_{\text{gain,r}}\text{-}\text{Q}_{\text{lose,r}}\text{-}\text{h}_{\text{r,air}}\text{(}\text{T}_{\text{r}}\text{-}\text{T}_{\text{air}}\text{)-}\text{U}_{\text{r,g}}\text{(}\text{T}_{\text{r}}\text{-}\text{T}_{\text{wg1}}\text{)=0}$ (1)

where,$\text{Q}_{\text{solar}}$ represents the solar radiation absorbed by the energy-saving film (W/m^2^). $\text{Q}_{\text{gain,r}}$ denotes atmospheric radiation absorbed by the film (W/m^2^), and $\text{Q}_{\text{lose,r}}$ refers to the heat lost through infrared emission (W/m^2^). $\text{h}_{\text{r,air}}$ is the convection heat transfer coefficient between the energy-saving film and outdoor air (W/(m^2^·K)). $\text{T}_{\text{r}}$ is the outer surface temperature of film (K), and $\text{T}_{\text{air}}$ is the outdoor air temperature (K), $\text{U}_{\text{r,g}}$ is the heat transfer coefficient of film (W/m^2^), and $\text{T}_{\text{wg1}}$ is the temperature on the outer surface of the glass (K).

$\text{Q}_{\text{solar}}$ can be calculated based on the solar irradiance and the spectral characteristics of the film, as shown in Eq. (2).

$\text{Q}_{\text{solar}}\text{=}\int_{\text{0}}^{\text{∞}} \text{d}\text{λ}\text{ ξ}_{\text{s}}\text{(}\text{λ}\text{) ×I}$ (2)

where $\text{ξ}_{\text{s}}\text{(}\text{λ}\text{)}$ represents the solar absorptivity of the energy-saving film, which can be measured using a UV-VIS-NIR spectrophotometer equipped with an integrating sphere.^[2]^ $\text{I}$ denotes the solar irradiance (W/m^2^).

The atmospheric radiation absorption and mid-infrared emission of the film can be calculated using Eq. (3) and Eq. (4) respectively.^[3,4]^

,$\text{Q}_{\text{gain,r}}\text{=2πA}\int_{\text{0}}^{\text{π/2}} \text{dθ sinθ cosθ}\int_{\text{0}}^{\text{∞}} \text{dλ }\text{I}_{\text{BB}}\text{ (}\text{T}_{\text{air}}\text{,λ) ε(λ,θ) }\text{ε}_{\text{a}}\text{(λ,θ) }$ (3)

$\text{Q}_{\text{lose,r}}\text{=2πA}\int_{\text{0}}^{\text{π/2}} \text{dθ sinθ cosθ}\int_{\text{0}}^{\text{∞}} \text{dλ }\text{I}_{\text{BB}}\text{ (}\text{T}_{\text{r}}\text{,λ) ε(λ,θ) }$ (4)

where $\text{I}_{\text{BB}}\text{ (}\text{T}\text{,}\text{ }\text{λ)}$ represents the spectral radiation of a black body at any wavelength $\lambda$, at the radiation temperature ($\text{T}_{\text{r}}$) or ambient temperature ($\text{T}_{\text{a}\text{ir}}$) according to Planck's law, and can be calculated using Eq. (5). $\text{ε(}\text{λ,θ}\text{)}$ denotes the emissivity of the film, while $\text{ }\text{ε}_{\text{a}}\text{(}\text{λ,θ}\text{)}$ represents the atmospheric emissivity, which can be calculated using Eqs. (6~8).^[5,6]^

$\text{I}_{\text{BB}}\text{ (}\text{T,λ}\text{)=}\frac{\text{2h}\text{c}^{\text{2}}}{\text{λ}^{\text{5}}}\frac{\text{1}}{\text{e}^{\text{hc}\text{/(λ}\text{k}_{\text{B}}\text{T)}}\text{-1}}$ (5)

$\text{ε}_{\text{a}}\text{(}\text{λ,θ}\text{)}\text{=ε(0)(1.4-0.4ε(0))(1+0.026C)}$ (6)

$\text{ε(0)=0.24+2.98×}\text{10}^{\text{-6}}\text{P}^{\text{2}}\text{exp(3000/}\text{T}_{\text{a}}\text{)}$ (7)

$\text{P=φ×}\text{P}_{\text{b}}$ (8)

where $\text{ε(}\text{0)}$ represents the horizontal atmospheric emission, $\text{ε}_{\text{a}}$ denotes the total atmospheric emission, and *C* is the cloud cover, ranging from 0-10, with 0 indicating a cloudless sky and 10 indicating fully overcast conditions.

$\text{h}_{\text{r,air}}\text{=2.8+3.0}\text{v}_{\text{air}}$ (9)

$\text{U}_{\text{r,g}}\text{=}\text{k}_{\text{r}}\text{/}\text{σ}_{\text{r}}$ (10)

where $\text{v}_{\text{air}}$ represents the outdoor wind speed (m/s), $\text{k}_{\text{r}}$ denotes the thermal conductivity of the film (W/(m·K)), and $\text{σ}_{\text{r}}$ is thickness of the film (m).

1. Thermal equilibrium of the outside surface of windows:

$\text{U}_{\text{r,g}}\text{(}\text{T}_{\text{r}}\text{-}\text{T}_{\text{wg1}}\text{)+}\text{Q}_{\text{solar,}\text{wg1}}\text{-}\text{U}_{\text{wg1}\text{,}\text{ng1}}\text{（}\text{T}_{\text{wg1}}\text{-}\text{T}_{\text{ng1}}\text{）}\text{-}\text{Q}_{\text{wg1}\text{,}\text{ng1}}\text{=0}$ (11)

where $\text{U}_{\text{wg1}\text{,}\text{ng1}}$ denotes the heat transfer coefficient of double glass (W/m^2^), $\text{T}_{\text{wg1}}$ and $\text{T}_{\text{ng1}}$ represent the temperature of the inside and outside surfaces of the window respectively (K). $\text{Q}_{\text{wg1}\text{,}\text{ng1}}$ refers to the radiant heat exchange between the inner and outer surfaces of window (W/m^2^), which can be calculated using Eq.(12). $\text{Q}_{\text{solar}\text{,}\text{wg1}}$ represents of the outer window in the double glass, as shown in Eq. (13).^[7]^

$\text{Q}_{\text{wg1,ng1}}\text{=5.67×}\text{10}^{\text{-8}}\text{×}\text{e}_{\text{g}}\text{×(}{\text{T}_{\text{wg1}}}^{\text{4}}\text{-}{\text{T}_{\text{n}\text{g1}}}^{\text{4}}\text{)}$ (12)

$\text{Q}_{\text{solar,}\text{wg1}}\text{=}\int_{\text{0}}^{\text{∞}} \text{(1-}\text{ ξ}_{\text{s}}\text{(λ)) ×}\text{ ξ}_{\text{g}}\text{×I }\text{dλ}$ (13)

where, $\text{e}_{\text{g}}$ is the mid-infrared emissivity of the glass, and $\text{ ξ}_{\text{g}}$ is the solar absorption rate of the glass.

1. Thermal equilibrium of the inner surface of the window:

$\text{Q}_{\text{solar,}\text{ng1}}\text{+}\text{U}_{\text{wg1}\text{,}\text{ng1}}\text{（}\text{T}_{\text{wg1}}\text{-}\text{T}_{\text{ng1}}\text{）}\text{+}\text{Q}_{\text{wg1}\text{,}\text{ng1}}\text{-}\text{h}_{\text{ng1,in }}\text{(}\text{T}_{\text{ng1}}\text{-}\text{T}_{\text{in}}\text{)+}\sum_{\text{i=1}}^{\text{n}} \text{J}_{\text{i, ng1}}\text{=0}$ (14)

where $\text{Q}_{\text{solar}\text{,}\text{n}\text{g1}}$ denotes the solar absorptivity of the glass (W/m^2^), $\text{h}_{\text{ng1,in }}$ represents the convection heat transfer coefficient between the window and the indoor air (W/(m^2^·K)), and $\text{J}_{\text{i, ng1}}$ denotes the long-wave radiative heat exchange from window $i$ (ceiling, floor, and interior walls) to window " $\text{ng1}\text{"}$. These values can be calculated using Eqs. (15~18) respectively.^[8,9]^

$\text{Q}_{\text{solar,}\text{ng1}}\text{ =}\int_{\text{0}}^{\text{∞}} \text{(1-}\text{ ξ}_{\text{s}}\text{(λ)) ×(1-}\text{ ξ}_{\text{g}}\text{)×}\text{ ξ}_{\text{g}}\text{×(}\text{I}_{\text{z}}\text{+}\text{I}_{\text{s}}\text{)}\text{dλ}$ (15)

$\text{h}_{\text{ng1,in }}\text{=}\text{N}_{\text{μ}}\text{×λ/x}$ (16)

$\text{J}_{\text{i, ng1}}\text{=}\frac{\text{E}_{\text{i}}\text{-}\text{E}_{\text{ng1}}}{\frac{\text{1-}\text{e}_{\text{i}}}{\text{A}_{\text{i}}\text{e}_{\text{i}}}\text{+}\frac{\text{1}}{\text{A}_{\text{i}}\text{X}_{\text{i,ng1}}}\text{+}\frac{\text{1-}\text{e}_{\text{ng1}}}{\text{A}_{\text{ng1}}\text{e}_{\text{ng1}}}}$ (17)

$\text{E}_{\text{i}}\text{=}\text{5.67×}\text{10}^{\text{-8}}\text{×}\text{e}_{\text{g}}\text{×}{\text{T}_{\text{i}}}^{\text{4}}$ (18)

where $N_{\mu}$ denotes the Nusselt number, $\text{λ}$ represents the thermal conductivity of the air, and $x$ is the characteristic length of the glass. $\text{E}_{\text{i}}$ and $\text{E}_{\text{ng1}}$ represent the blackbody radiation of wall *i* and the glass, respectively. $\text{e}_{\text{i}}$ and $\text{e}_{\text{ng1}}$ denote the emissivity of the wall and the glass, respectively. $\text{A}_{\text{i}}$ and $\text{A}_{\text{ng1}}$ represent the effective areas of wall *i* and glass (m^2^). $\text{X}_{\text{i,ng1}}$ is the angle factor between wall *i* and glass. $\text{T}_{\text{i}}$ is the surface temperature of the wall *i* (K).

1. Thermal equilibrium of the building’s interior wall:

$\sum_{\text{i=1}}^{\text{n}} \text{J}_{\text{i, j}}\text{+}\text{J}_{\text{ng1, j}}\text{-}\text{h}_{\text{j,in}}\text{(}\text{T}_{\text{j}}\text{-}\text{T}_{\text{in}}\text{)+}\text{Q}_{\text{solar, j}}\text{=0}$ (19)

where $\sum_{\text{i=1}}^{\text{n}} \text{J}_{\text{i, j}}$ represents the long-wave radiative heat exchange from window $i$ (ceiling, floor and interior walls) to wall $j$, which can also be calculated using Eq. (17). $\text{Q}_{\text{solar}\text{, j}}$ denotes the solar radiation absorbed by the wall $j$ (W/m^2^), as described in Eq. (20).

$\text{Q}_{\text{solar, j}}\text{ =}\int_{\text{0}}^{\text{∞}} \text{(1-}\text{ ξ}_{\text{s}}\text{(}\text{λ}\text{)}\text{)}\text{ }\text{×}{\text{(1-}\text{ ξ}_{\text{g}}\text{)}}^{\text{2}}\text{×}\text{ ξ}_{\text{j}}\text{×I}\text{×}\text{X}_{\text{ng1,j}}\text{d}\text{λ}$ (20)

where$\text{ ξ}_{j}$ represents the solar absorption rate of the wall $j$.

1. Thermal equilibrium of the inner surface of the building’s exterior wall:

$\sum_{\text{i=1}}^{\text{n}} \text{J}_{\text{i, j}}\text{+}\text{U}_{\text{w}}\text{(}\text{T}_{\text{w}}\text{-}\text{T}_{\text{j}}\text{)-}\text{h}_{\text{j,in}}\text{(}\text{T}_{\text{j}}\text{-}\text{T}_{\text{in}}\text{)=0}$ (21)

where $\text{U}_{\text{w}}$ represents the thermal conductivity of the wall (W/(m^2^·K)), and $\text{T}_{\text{j}}$and $\text{T}_{\text{w}}$ are the temperature of the inner and outer surfaces (K).

1. Thermal balance of the outer surface of building's exterior wall:

$\text{Q}_{\text{solar,w}}\text{+}\text{Q}_{\text{gain,w}}\text{-}\text{Q}_{\text{lose,w}}\text{-}\text{h}_{\text{w,air}}\text{(}\text{T}_{\text{w}}\text{-}\text{T}_{\text{air}}\text{)-}\text{U}_{\text{w}}\text{(}\text{T}_{\text{w}}\text{-}\text{T}_{\text{j}}\text{)=0}$ (22)

where $\text{Q}_{\text{solar}\text{,w}}$ denotes the solar radiation absorbed by exterior wall (W/m^2^), $\text{Q}_{\text{gain,w}}$ represents the atmospheric radiation absorbed by the exterior wall (W/m^2^), $\text{Q}_{\text{lose,w}}$ refers to the heat lost through infrared emission (W/m^2^), and $\text{h}_{\text{w,air}}$ represents the convection heat transfer coefficient between the exterior wall and outdoor air (W/(m^2^·K)). These parameters can be calculated separately using Eqs. (2~10).

1. Thermal balance of indoor air:

$\sum_{\text{j=1}}^{\text{n}} \text{h}_{\text{j,in}}\text{(}\text{T}_{\text{j}}\text{-}\text{T}_{\text{in}}\text{)+}\text{h}_{\text{ng1,in }}\text{(}\text{T}_{\text{ng1}}\text{-}\text{T}_{\text{in}}\text{)+}\text{Q}_{\text{HVAC}}\text{+}\text{Q}_{\text{light}}\text{+}\text{Q}_{\text{air}}\text{+}\text{Q}_{\text{solar,in}}\text{=0}$ (23)

where $\text{Q}_{\text{HVAC}}$ denotes the cooling capacity or heating capacity of the air conditioner (W). $\text{Q}_{\text{light}}$ represents the heat from the lighting (W).^[10]^ $\text{Q}_{\text{air}}$ refers to the fresh air load (W), and $\text{Q}_{\text{solar}\text{,in}}$ represents the solar radiation absorbed in the room (W), which can be calculated using Eq (24) and Eq (25).

$\text{Q}_{\text{air}}\text{=}\text{c}_{\text{p}}\text{×30n (}\text{T}_{\text{air}}\text{-}\text{T}_{\text{in}}\text{)}$ (24)

$\text{Q}_{\text{solar, j}}\text{ =}\int_{\text{0}}^{\text{∞}} \text{(1-}\text{ ξ}_{\text{s}}\text{(}\text{λ}\text{)}\text{)}\text{ }\text{×}{\text{(1-}\text{ ξ}_{\text{g}}\text{)}}^{\text{2}}\text{×}\text{I }\text{d}\text{λ}$ (25)

where$\text{n}$ represents the number of people in the room. $\text{c}_{\text{p}}$ is the specific heat capacity of air (J/ (kg·K)).

The energy consumption of the air conditioning can be calculated by maintaining $\text{T}_{\text{in}}$ as a constant when the air conditioner is operating.

Energy-efficient films, particularly thermochromic films, become opaque at high temperatures, reducing cooling power consumption but potentially increasing lighting energy consumption significantly. Therefore, in this study, lighting energy consumption was calculated based on lighting demand. The luminance of solar irradiation through the window can be calculated using Eq. (26).^[11]^

$\text{Lux}_{\text{sun}}\text{ =0.033}{\text{Q}_{\text{solar,in}}}^{\text{3}}\text{-0.3381}{\text{Q}_{\text{solar,in}}}^{\text{2}}\text{+212.94}\text{Q}_{\text{solar,in}}\text{-768.01}$ (26)

The luminaire needs to be turned on when the illumination provided by solar irradiation is less than the lighting demand, and the energy consumption can be calculated by Eq. (27).

$\text{P}_{\text{light}}\text{=(}\text{Lux}_{\text{0}}\text{-}\text{Lux}_{\text{sun}}\text{)/φ/A}$ (27)

where $\text{P}_{\text{light}}$ represents the lighting energy consumption per unit area (W/m^2^), $\text{Lux}_{\text{0}}$ denotes indoor lighting demand (lux), $\text{Lux}_{\text{sun}}$ is the illumination provided by solar irradiation (lux), $\text{φ}$ is the conversion between energy consumption and illumination of LED lamp (70~90 lux/W).^[12]^ *A* is the interior area (m^2^).

# Supplementary Figures

**Figure S1. Fourier transform infrared spectrum information of solar HPMC-borax hydrogel.**


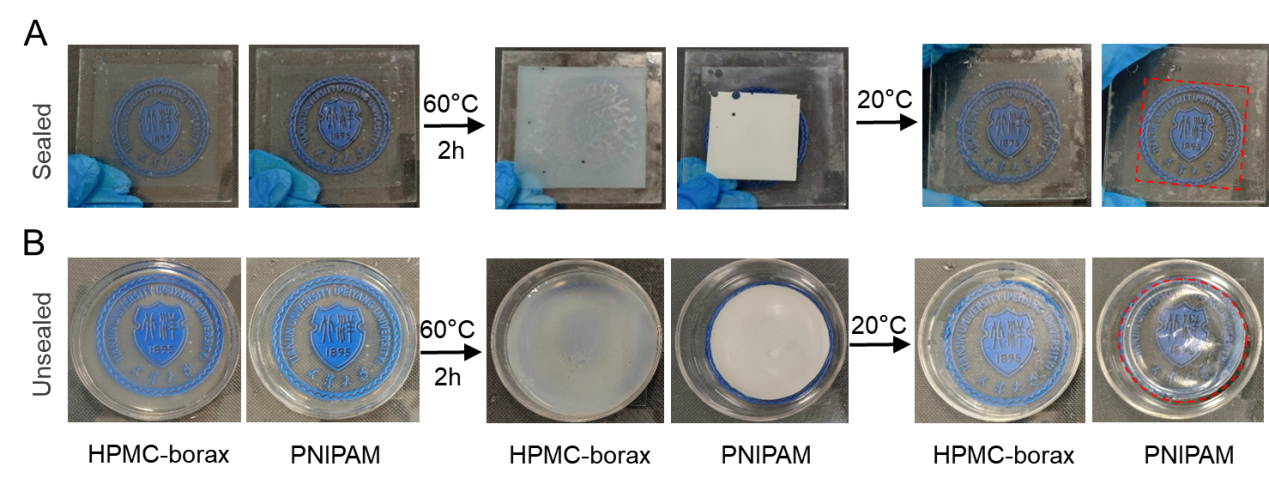


**Figure S2. HPMC-borax hydrogel shrinkage resistance under sealed and unsealed conditions.**

**
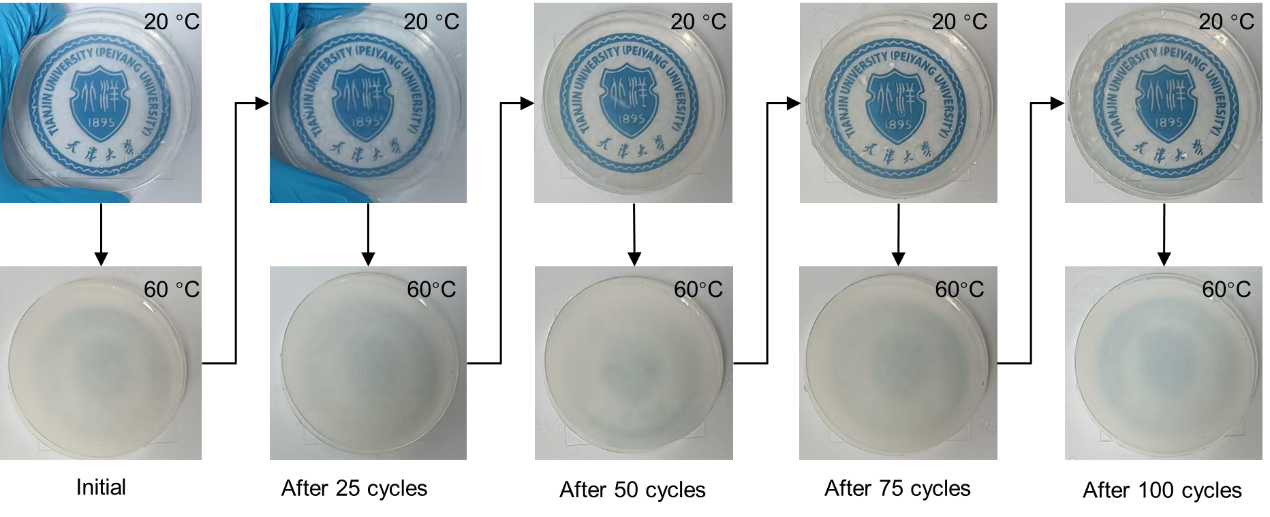
**

**Figure S3. Thermal cycling stability of the HPMC-borax hydrogel.**


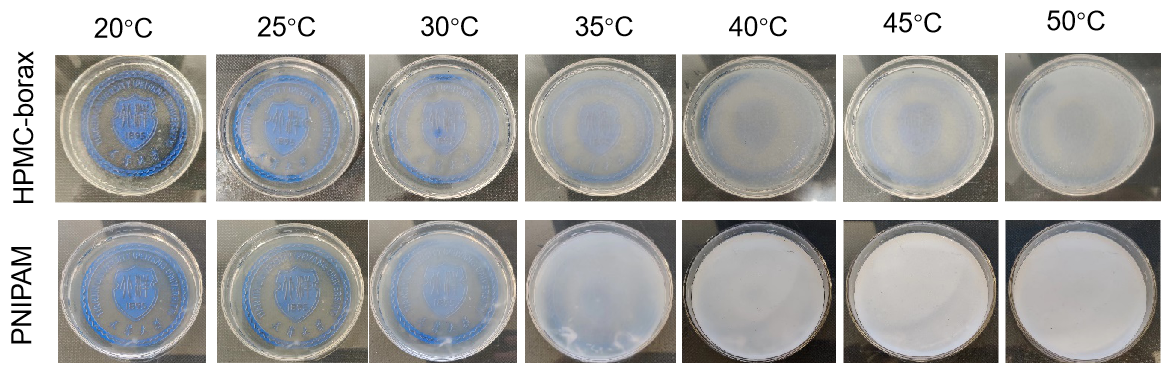


**Figure S4. Thermochromic process of HPMC-borax and PNIPAM hydrogel.**


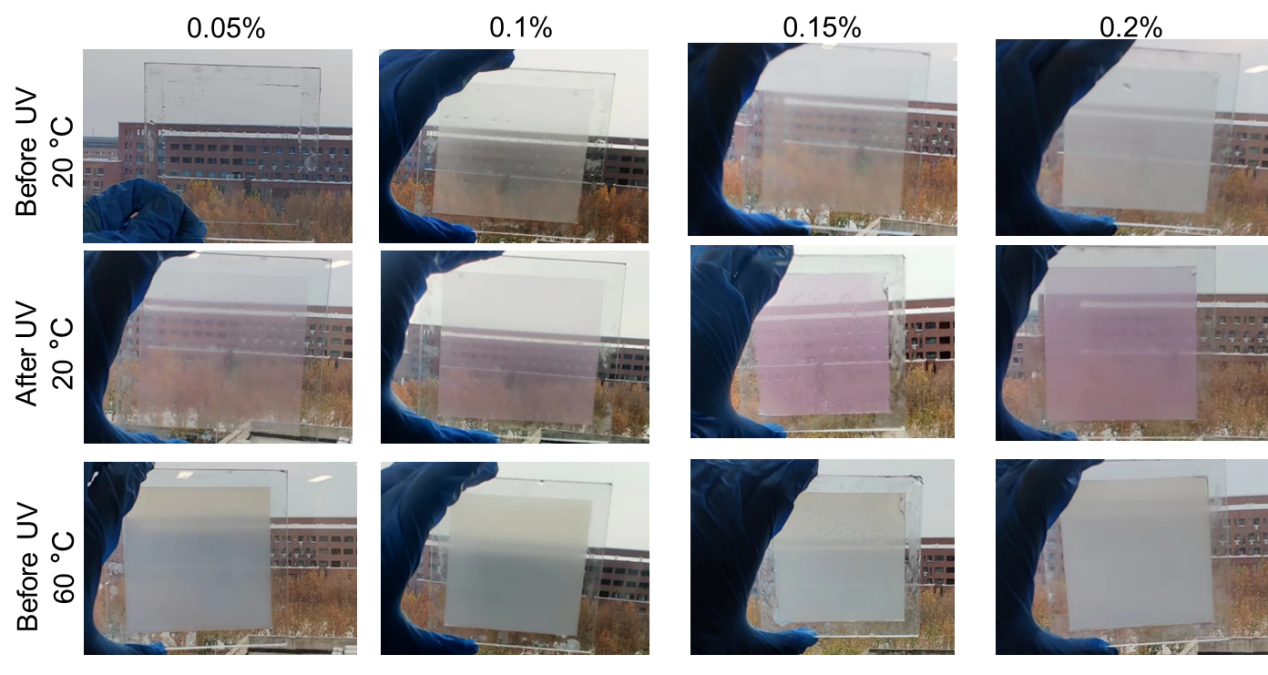


**Figure S5. The color change effect of AMDTR smart window after adding red photochromic powder to hydrogel.**


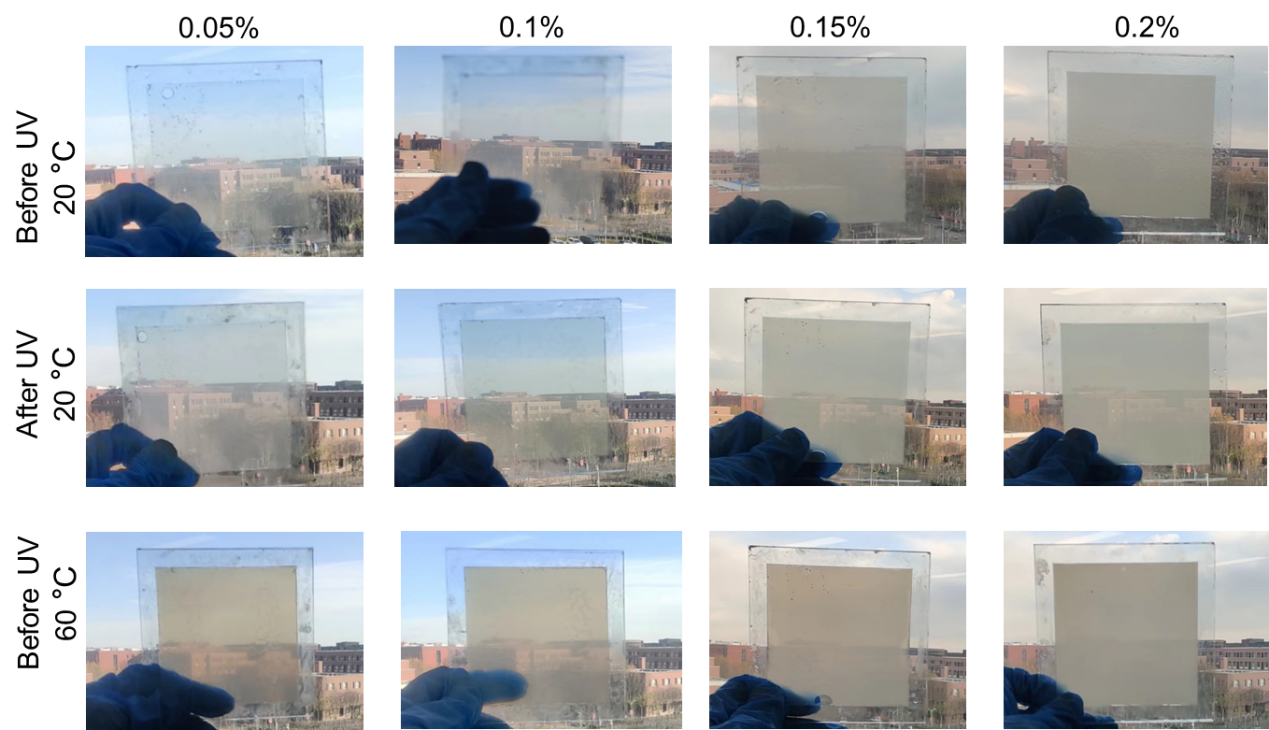


**Figure S6. The color change effect of AMDTR smart window after adding green photochromic powder to hydrogel.**


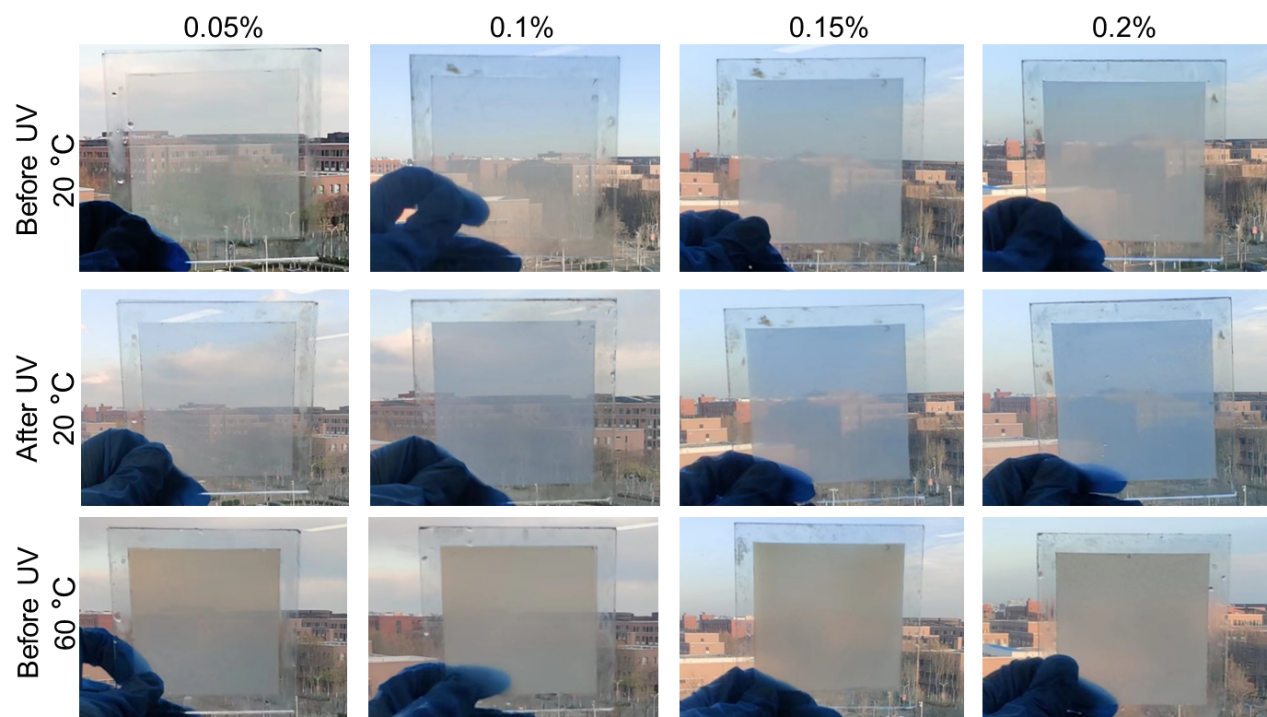


**Figure S7. The color change effect of AMDTR smart window after adding blue photochromic powder to hydrogel.**

**
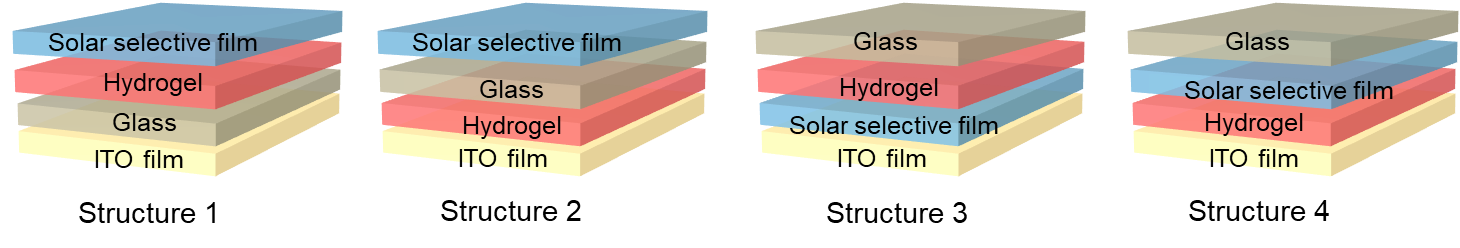
**

**Figure S8. Different** **structurals of AMDTR smart windows.**

**
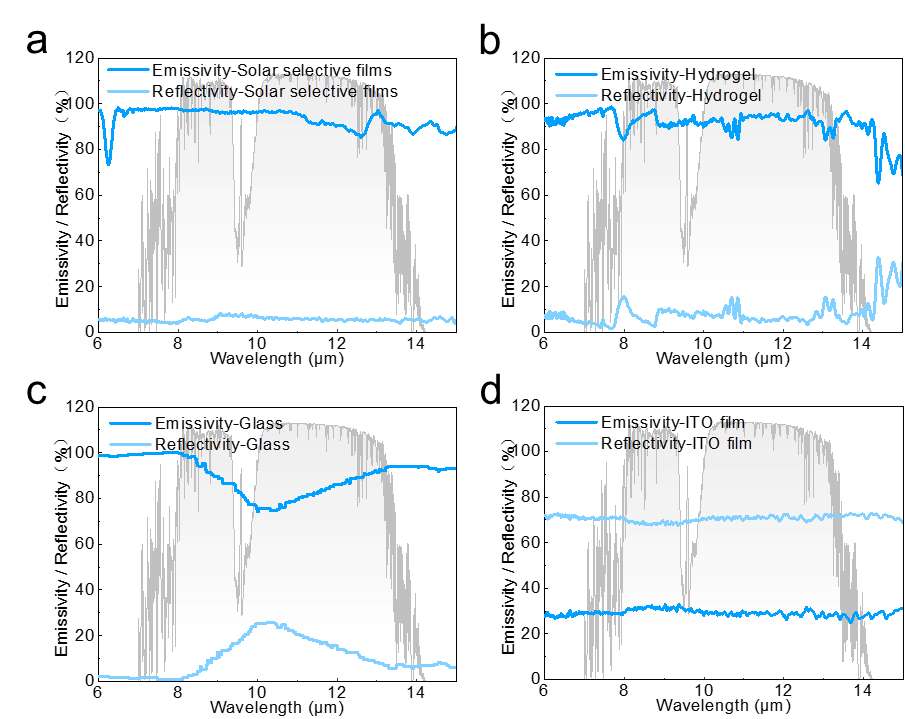
**

**Figure S9. The emissivity and reflectivity of solar selective film (a), hydrogel (b), glass (c) and ITO film (d).**

**
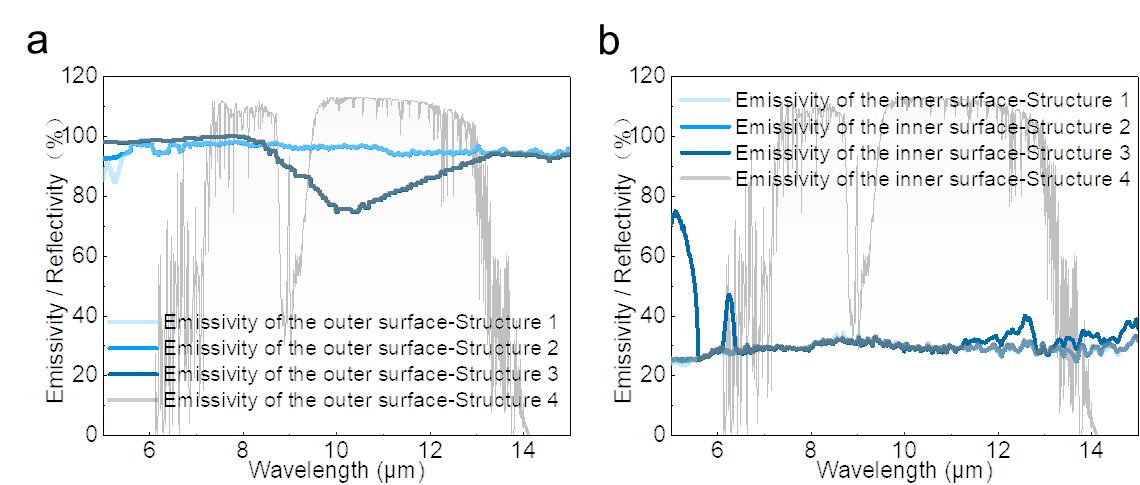
**

**Figure S10. The emissivity of the outer(a) and inner (b) surfaces of the AMDTR smart windows with different structures.**

**
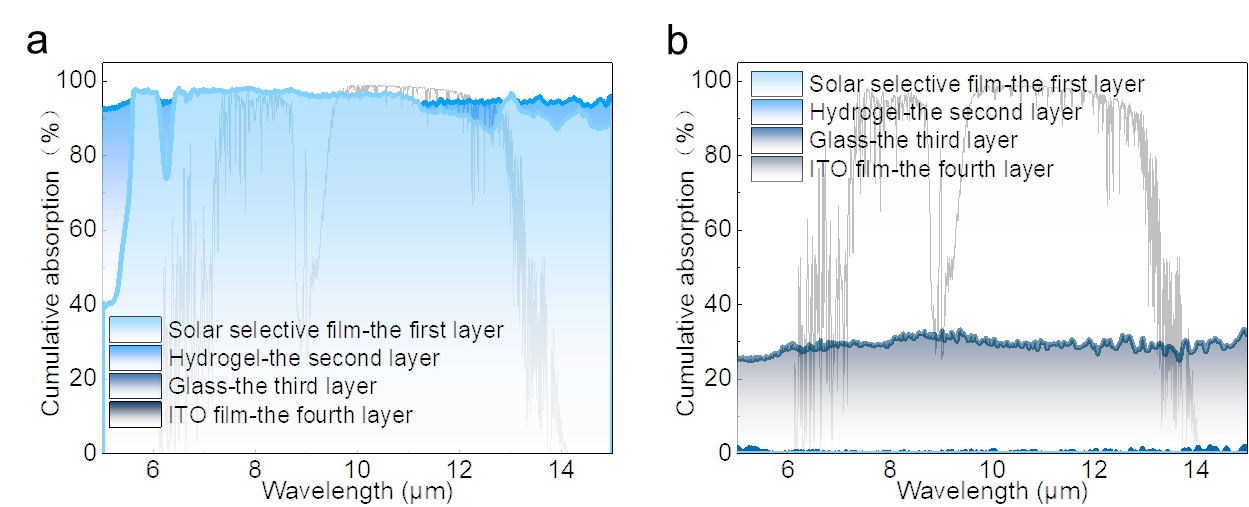
**

**Figure S11. The emissivity contribution of each functional layer in AMDTR smart window (structure 1) to outer (a) and inner (b) surfaces.**

**
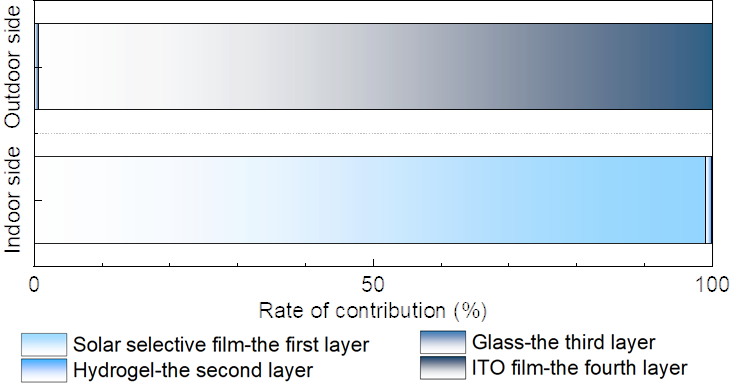
**

**Figure S12. The contribution rate of each functional layer in AMDTR smart window to outer (a) and inner (b) surfaces.**


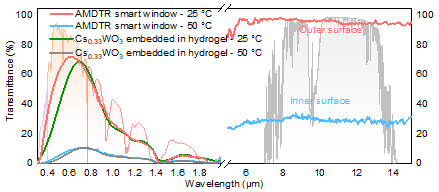


**Figure S13. Spectral comparison of Cs_0.33_WO_3_ nanoparticles directly embedded in hydrogel and AMDTR smart window.**

**
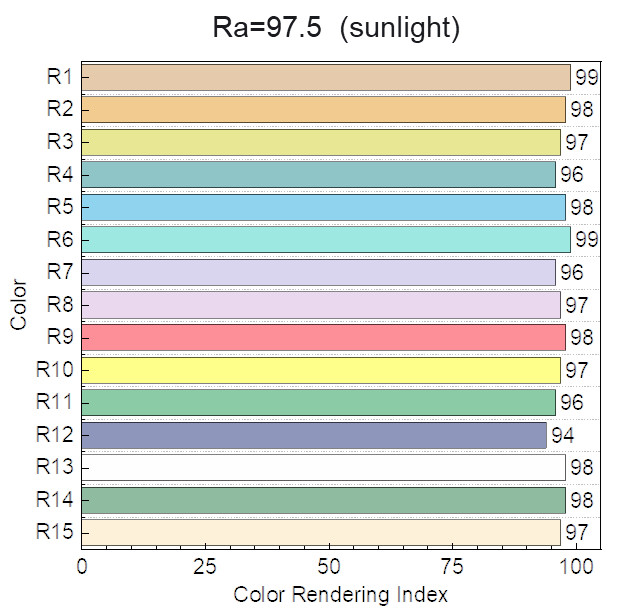
**

**Figure S14. Color Rendering Index of natural light.**

**
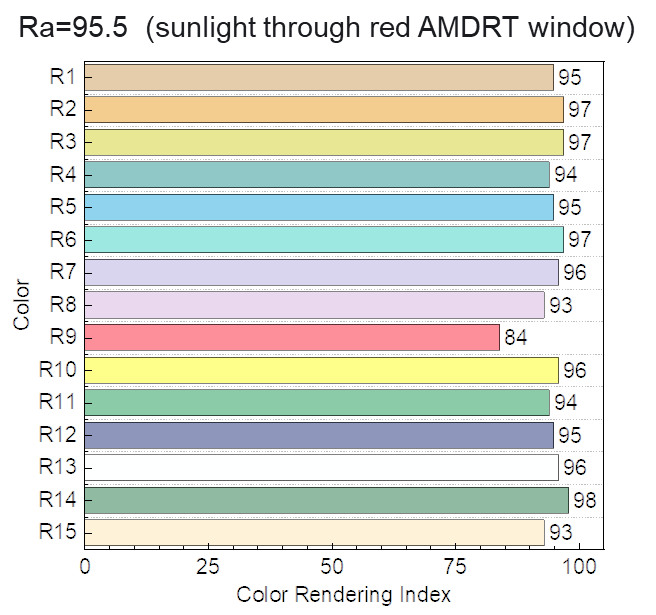
**

**Figure S15. Color rendering index of sunlight transmitted through the pink AMDRT smart window containing pink photochromic powder.**

**
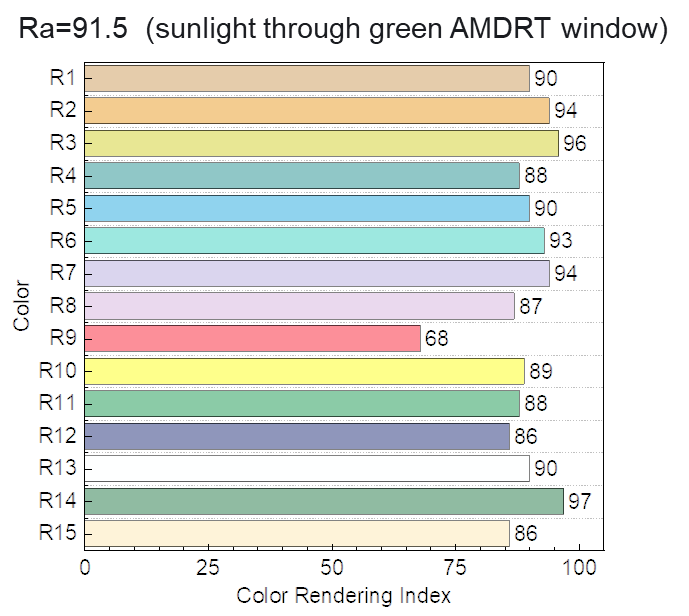
**

**Figure S16. Color rendering index of sunlight transmitted through the green AMDRT smart window.**

**
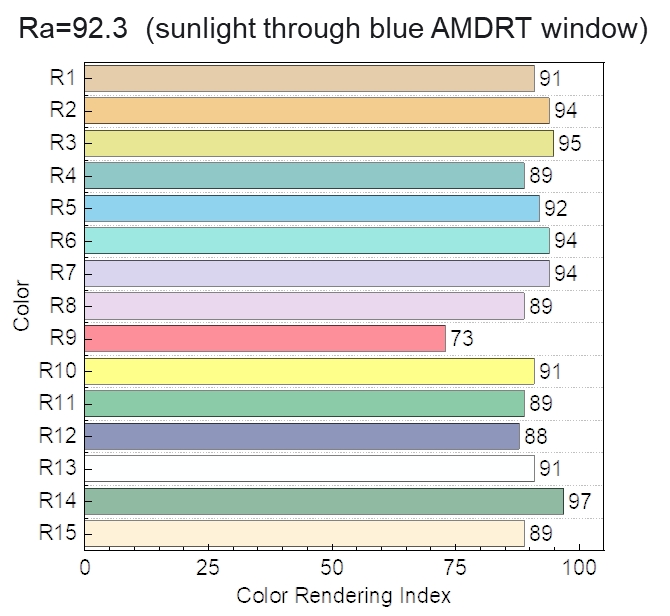
**

**Figure S17. Color rendering index of sunlight transmitted through the blue AMDRT smart window.**

**
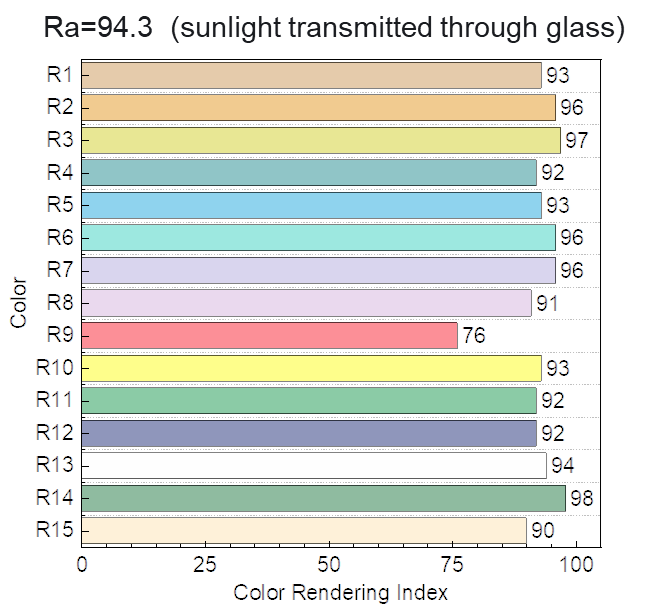
**

**Figure S18. Color rendering index of sunlight transmitted through the glass.**

**
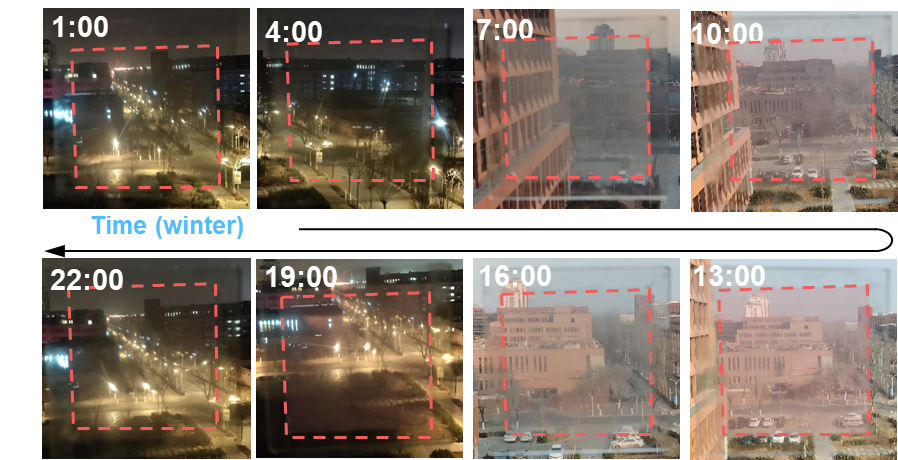
**

**Figure S19.** **The color change process of AMDTR smart windows throughout an entire day in winter.**

**
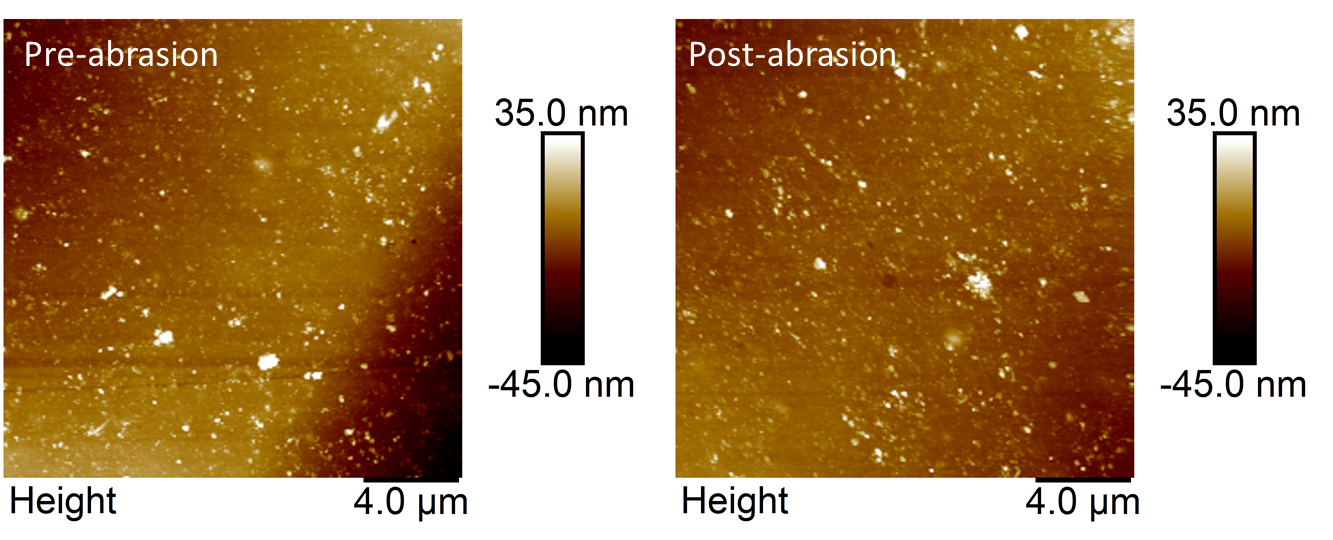
**

**Figure S20. Atomic force microscope images before and after abrasion.**

**
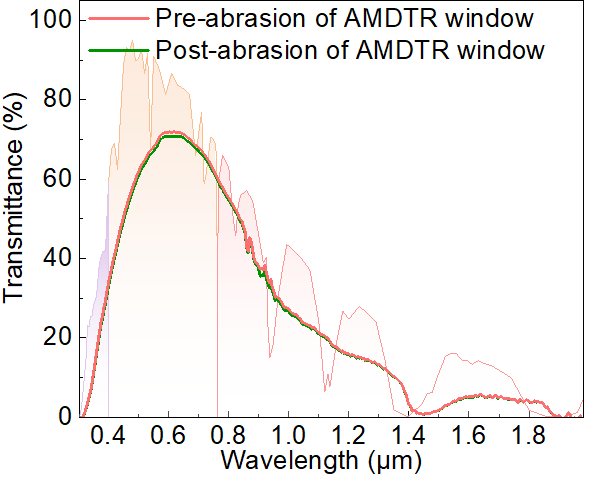
**

**Figure S21. Optical properties of the AMDTR smart windows before and after abrasion.**

**
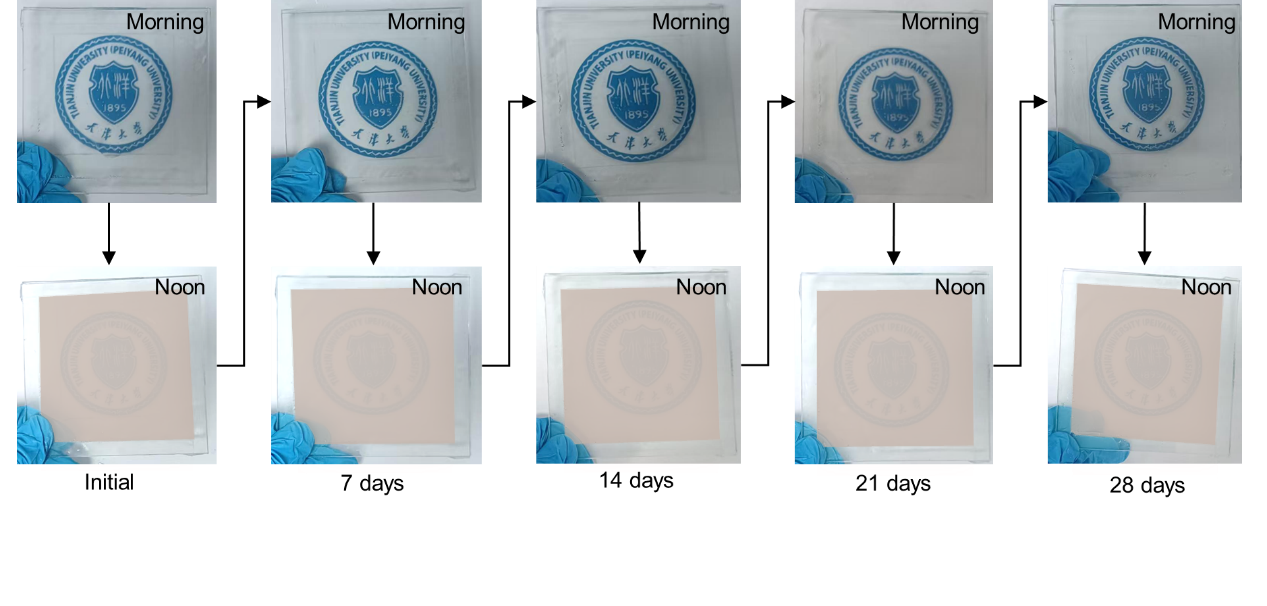
**

**Figure S22. The color change process of AMDTR smart windows within one month.**


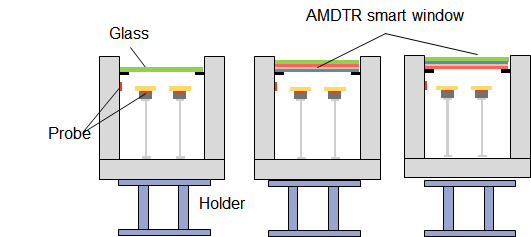


**Figure S23. Schematic diagram of experimental apparatus.**


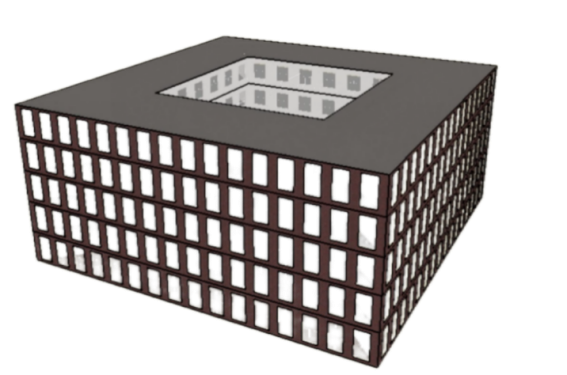


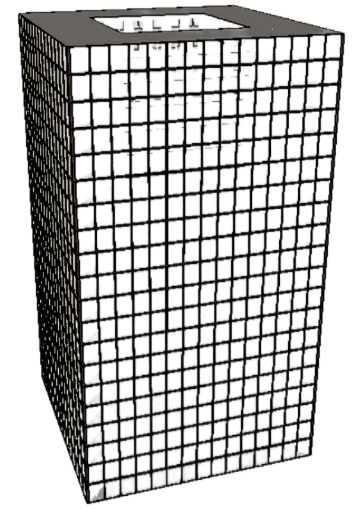


**Figure S24. Simulation model of the normal building and glass curtain wall building.**

**Figure S25. Indirect temperature drop comparison between AMDTR smart window and solar selective film.**

**Figure S26. Indirect temperature drop comparison between AMDTR smart window and PNIPAM hydrogel.**

**Figure S27. Indirect temperature drop when the high-emissivity side (solar selective film) and low-emissivity side (ITO film) were oriented outward.**


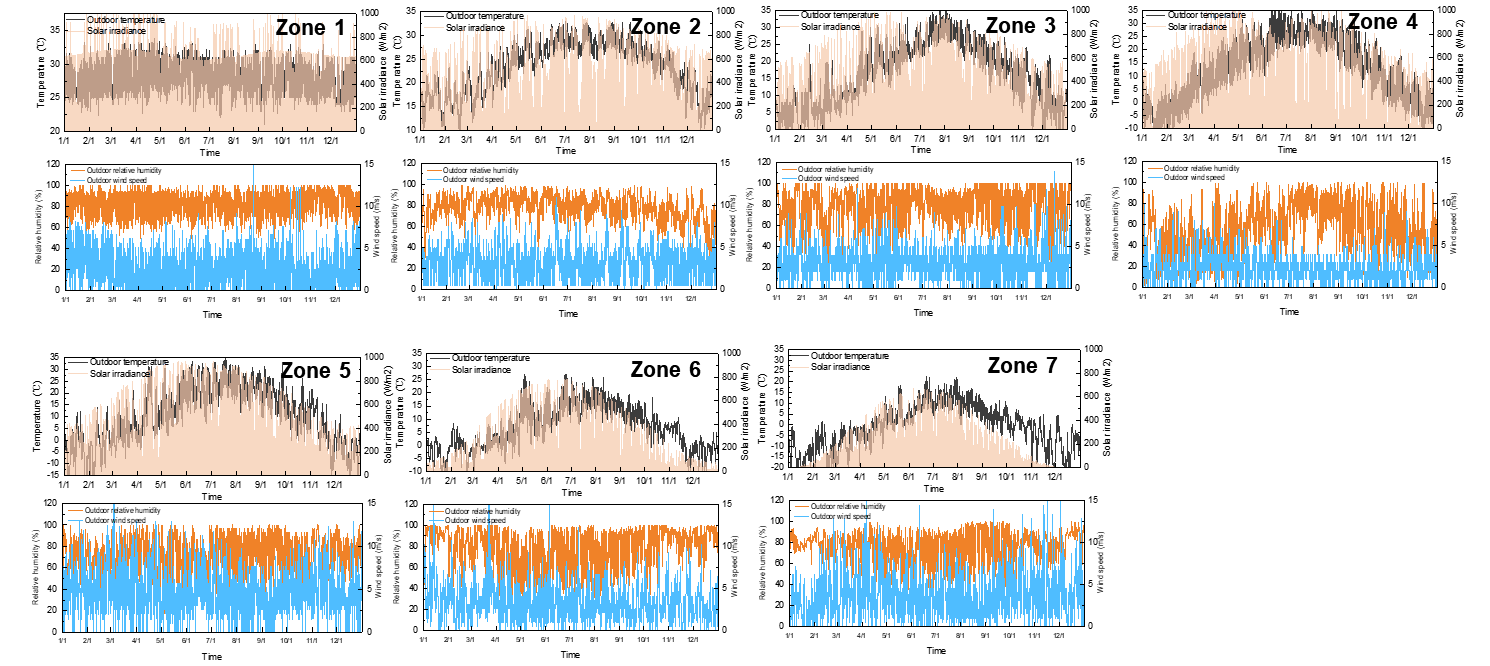


**Figure S28. Annual meteorological parameters during energy consumption simulation in different regions.**


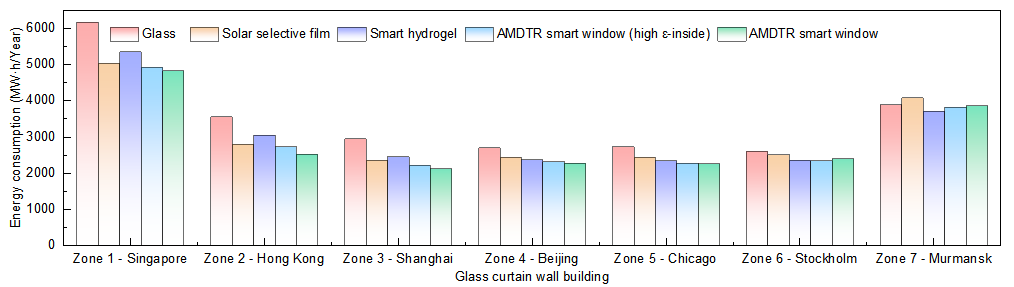


**Figure S29. Annual energy savings of different energy-saving window technologies for glass curtain wall buildings.**

# Supplementary Tables

**Table S1**. The emissivity of each functional layer.

| Structure | Emissivity | Reflectivity |
| --- | --- | --- |
| Solar selective film | **94.8%** | 5.1% |
| Hydrogel | 92.5% | 7.4% |
| Glass | 85.6% | 14.3% |
| ITO | 30.0% | **68.8%** |

**Table S2**. The emissivity of AMDTR smart Windows with different structures.

| Structure | | Outer surface | | Inner surface | | Emissivity difference (Δε) |
| --- | --- | --- | --- | --- | --- | --- |
|  |  | Emissivity | Reflectivity | Emissivity | Reflectivity |  |
| 1 | Solar selective film/ hydrogel / glass /ITO | **96.1%** | **3.8%** | **30.1%** | **69.8%** | **66.0%** |
| 2 | Solar selective film / glass / hydrogel /ITO | 96.0% | 3.9% | 30.2% | 69.7% | 65.8% |
| 3 | Glass / hydrogel / solar selective film / ITO | 86.3% | 13.6% | 31.0% | 68.9% | 55.3% |
| 4 | Glass / solar selective film / hydrogel / ITO | 86.3% | 13.6% | 30.2% | 69.7% | 56.1% |

**Table S3**. Basic building parameters in energy consumption analysis.

| Parameter | Normal building | Glass-curtain building |
| --- | --- | --- |
| Number of floors | 5 | 20 |
| Window-wall ratio of the exterior window | 50% | 90% |
| Type of building | Office building | |
| Thermal conductivity of exterior wall | 0.5 W/(m^2^·K) | |
| Thermal conductivity of roof | 0.35 W/(m^2^·K) | |
| Covers an area | 50*50 m | |
| Type of glass | Double glazing unit (6-12A-6) | |
| Window wall ratio of the inner window | 30% | |
| The Windows and walls on the roof | 25% | |
| Lighting demand | 300 lux/m^2^ | |
| Room temperature setting range | 20~25 ℃ | |
| Air conditioner opening period | Weekdays: 8:30~17:30 | |
| COP of air conditioner | 3 | |
| Application of solar selective film | External walls and roof Windows (except north in the Northern Hemisphere or south in the Southern Hemisphere) | |

# References

[1] C. Wang, H. Wei, Z. Zhou, Y. Chao, J. Liu, X. Yang, Y. Du, W. Wang, L. Yu, S. Zhang, J. Yan, *J. Mater. Chem. A* **2024**, 2208.

[2] Y. Fu, J. Yang, Y. S. Su, W. Du, Y. G. Ma, *Sol. Energy Mater. Sol. Cells* **2019**, *191*, 50.

[3] M. M. Hossain, M. Gu, *Adv. Sci.* **2016**, *3*, 1500360.

[4] J. Liu, Z. Zhou, J. Zhang, W. Feng, J. Zuo, *Mater. Today Phys.* **2019**, *11*, 100161.

[5] M. Li, Y. Jiang, C. F. M. Coimbra, *Sol. Energy* **2017**, *144*, 40.

[6] C. Liu, Y. Wu, B. Wang, C. Y. Zhao, H. Bao, *Sol. Energy* **2019**, *183*, 218.

[7] T. T. Chow, *Sol. Energy* **2003**, *75*, 143.

[8] Y. Tang, Q. Tao, Y. Chen, J. Zheng, Y. Min, *J. Build. Eng.* **2023**, *74*, 106869.

[9] M. Camci, Y. Karakoyun, O. Acikgoz, A. S. Dalkilic, *Energy Build.* **2021**, *242*, 110985.

[10] I. Din, H. Kim, *IEEE Photonics Technol. Lett.* **2014**, *26*, 781.

[11] W. Yao, Z. Li, C. Li, Z. Ai, *Tongji Daxue Xuebao/Journal Tongji Univ.* **2013**, *41*, 784.

[12] J. A. Mathias, K. M. Juenger, J. J. Horton, *Energy Effic.* **2023**, *16*, 34.
